# Characterization of viroplasm-like structures by co-expression of NSP5 and NSP2 across rotavirus species A to J

**DOI:** 10.1128/jvi.00975-24

**Published:** 2024-08-28

**Authors:** Melissa Lee, Ariana Cosic, Kurt Tobler, Claudio Aguilar, Cornel Fraefel, Catherine Eichwald

**Affiliations:** 1 Institute of Virology, University of Zurich, Zurich, Switzerland; University of Michigan Medical School, Ann Arbor, Michigan, USA

**Keywords:** rotavirus, species, viroplasm, NSP5, NSP2, oligomerization, tail, viroplasm-like structures, NSP5 C-terminus

## Abstract

Rotaviruses (RVs) are classified into nine species, A–D and F–J, with species A being the most studied. In rotavirus of species A (RVA), replication occurs in viroplasms, which are cytosolic globular inclusions composed of main building block proteins NSP5, NSP2, and VP2. The co-expression of NSP5 with either NSP2 or VP2 in uninfected cells leads to the formation of viroplasm-like structures (VLSs). Although morphologically identical to viroplasms, VLSs do not produce viral progeny but serve as excellent tools for studying complex viroplasms. A knowledge gap exists regarding non-RVA viroplasms due to the lack of specific antibodies and suitable cell culture systems. In this study, we explored the ability of NSP5 and NSP2 from non-RVA species to form VLSs. The co-expression of these two proteins led to globular VLSs in RV species A, B, D, F, G, and I, while RVC formed filamentous VLSs. The co-expression of NSP5 and NSP2 of RV species H and J did not result in VLS formation. Interestingly, NSP5 of all RV species self-oligomerizes, with the ordered C-terminal region, termed the tail, being necessary for self-oligomerization of RV species A–C and G–J. Except for NSP5 from RVJ, all NSP5 interacted with their cognate NSP2. We also found that interspecies VLS are formed between closely related RV species B with G and D with F. Additionally, VLS from RVH and RVJ formed when the tail of NSP5 RVH and RVJ was replaced by the tail of NSP5 from RVA and co-expressed with their respective NSP2.

**IMPORTANCE:**

Rotaviruses (RVs) are classified into nine species, A–D and F–J, infecting mammals and birds. Due to the lack of research tools, all cumulative knowledge on RV replication is based on RV species A (RVA). The RV replication compartments are globular cytosolic structures named viroplasms, which have only been identified in RV species A. In this study, we examined the formation of viroplasm-like structures (VLSs) by the co-expression of NSP5 with NSP2 across RV species A to J. Globular VLSs formed for RV species A, B, D, F, G, and I, while RV species C formed filamentous structures. The RV species H and J did not form VLS with their cognates NSP5 and NSP2. Similar to RVA, NSP5 self-oligomerizes in all RV species, which is required for VLS formation. This study provides basic knowledge of the non-RVA replication mechanisms, which could help develop strategies to halt virus infection across RV species.

## INTRODUCTION

Rotavirus (RV) is an etiological agent responsible for severe gastroenteritis in infants and young children, killing approximately 128,000 children per year, mainly in low- and middle-income countries (1). Additionally, RV outbreaks are the leading cause of diarrhea among the adult population in several countries (2–6). In the United States, RV infection ranks as the second most common cause of diarrhea in adults after norovirus (7–9). From a veterinary perspective, RV infections significantly impact livestock worldwide. RV accounts for 80% of diarrhea cases in piglets in the United States, Canada, and Mexico, with potential zoonotic implications in humans (10). In the poultry industry, RV infections impact the feed conversion ratio, resulting in substantial economic losses (11).

RV of species A (RVA) is a segmented double-stranded RNA (dsRNA) virus belonging to the *Reoviridae* family. The RV virion is organized in three concentric layers surrounding the viral genome. The spike protein VP4 and the glycoprotein VP7 compose the outermost layer, while VP6 forms the intermediate layer. The innermost layer, the core-shell, comprises 120 copies of VP2, organized in 12 asymmetric decamers (12). Each core-shell encapsidates 11 genomic RNA segments, and the replication are complexes composed of the RNA-dependent RNA polymerase VP1 and the guanylylmethyltransferase VP3, which localize beneath each of the fivefold axes of the VP2 decamer. The external layer is lost during virus entry, and a transcriptionally active double-layered particle is released into the cytosol (13). The newly released transcripts are translated into viral proteins necessary for viral replication. Among those proteins, the NTPase/RTPase NSP2 and the phosphoprotein NSP5, together with the structural proteins VP1, VP2, VP3, and VP6, make part of the RV replication compartments termed viroplasms (14). The viroplasms correspond to electron-dense, membrane-less globular cytosolic inclusions where viral genome transcription, replication, and the packaging of the newly synthesized pre-genomic RNA segments into the viral cores occur. The viroplasms are highly dynamic, being able to coalesce between them and move to the juxtanuclear region of the cell at increasing times post-infection (15–17). Despite not yet being well-defined, several host factors have been identified as necessary for viroplasm formation and maintenance (18–21). The initiation process for viroplasm formation requires a scaffold of lipid droplets by incorporating perilipin-1 (22, 23). Furthermore, the host cytoskeleton, actin filaments, and microtubules (MTs) play a role in the formation, maintenance, and dynamics of the viroplasms (16, 24, 25). In this context, NSP2 octamers are directly associated with MTs to promote viroplasm coalescence (16, 26–29). Moreover, VP2 plays a role in viroplasm dynamics by allowing their perinuclear motion (16). Finally, consistent with these features, the viroplasms were found to be subject to liquid-liquid phase-separated structures (30). Interestingly, co-expression of the main viroplasm protein NSP5 with either NSP2 or VP2 leads to the formation of cytosolic inclusions named viroplasm-like structures (VLSs), which are morphologically similar to viroplasms but unable to yield viral progeny (15, 16, 31–34).

NSP5 is required for viroplasm formation and virus replication (35–37), having a multifunctional role in the RV life cycle, interacting with NSP6 (34), NSP2 (15), VP1 (38), VP2 (39, 40), and unspecifically to dsRNA (41). These attributes are consistent with the predicted flexible structure of NSP5 (42–44). Interestingly, the C-terminal region of NSP5 is needed for its self-oligomerization (34, 45), to associate with other RVA proteins (15, 34, 38, 40), and to form the viroplasms (37). NSP5 is sumoylated (46), presumably a pre-requirement for interacting with viral or host components. NSP5 is also phosphorylated, which is crucial for viroplasm morphology (37), a trait for liquid-liquid phase separation conditions of the viroplasms (30). NSP5 hyperphosphorylation is triggered by the association with NSP2 or VP2, primed at serine-67 by casein kinase 1 alpha (31, 45, 47, 48). Collectively, NSP5 is a crucial component in RVA replication.

NSP2 self-assembles in two doughnut-shaped tetramers with a highly electropositive nature (27, 49). NSP2 has several enzymatic activities, such as nucleoside diphosphate kinase-like (50), RNA-helix-destabilizing (50), and nucleoside triphosphatase activities (27) required for RV genome replication. Also, NSP2 phosphorylation has been implicated in viroplasm formation, as evidenced by the delayed formation of viroplasms observed in an NSP2 S313D phosphomimetic mutant (22). NSP2 directly influences viroplasm coalescence (15) through association with MTs (28). The flexible NSP2 C-terminus also improves viroplasm morphology (51) and its activity as an RNA chaperone (26). Interestingly, NSP2 binds to VP1 and viral RNA (52, 53), implicating it as a factor in replication intermediates in the viroplasms.

All the current information compiled on the RV replication life cycle is based on species A data, which has a broad spectrum of strains that mainly infect young mammals like infants, piglets, and calves. According to the International Committee on Taxonomy of Viruses (ICTVs), RVs are currently grouped into nine species (54): A to D and F to J. Although not fully recognized yet, it should be noted that there is evidence for at least two additional RV species, K (55, 56) and L (57). These species have been detected in diverse hosts. Rotavirus B (RVB) has been identified in human adults, rats, cattle, goats, sheep, and swine (58). RVs D (RVD), F (RVF), and G (RVG) have been only detected in avian species. Studying the replication of these RV species is challenging since most of the knowledge about them has been obtained by in-depth sequencing of nucleic acid isolated from infected samples, and therefore, no virus inoculum is available for their investigation. The few isolated viruses of non-RVA species are not well adapted to tissue culture (59), and research tools such as specific antibodies are unavailable. Therefore, all information about RV replication and life cycle is derived from RVA species. For non-RVA species, there is no evidence for viroplasm formation, which is crucial for virus replication. A comprehensive approach to the mechanism of replication of non-RVA species is essential for tackling RV infection in diverse hosts, including humans (RVB), pigs (RVA and RVC), and avians (RVD, RVF, and RVG). RV reverse genetics has been recently established only for some RVA strains like simian SA11 (60), porcine OSU (61), and human KU (62). Therefore, using this technology for other RV species is still unfeasible.

In this study, we investigated whether RV species B to J can form globular VLS by co-expressing their corresponding NSP5 and NSP2 proteins. We demonstrate that NSP5 in all tested RV species can self-oligomerize, primarily through their predicted structurally ordered region. Additionally, we tested the ability of NSP5 to bind NSP2 in non-RVA species. Finally, we examined RV interspecies formation of VLS and found that some RV species can form heterologous NSP5- and NSP2-induced VLS.

## RESULTS

### NSP5 and NSP2 across species A to J

**Fig 1:**
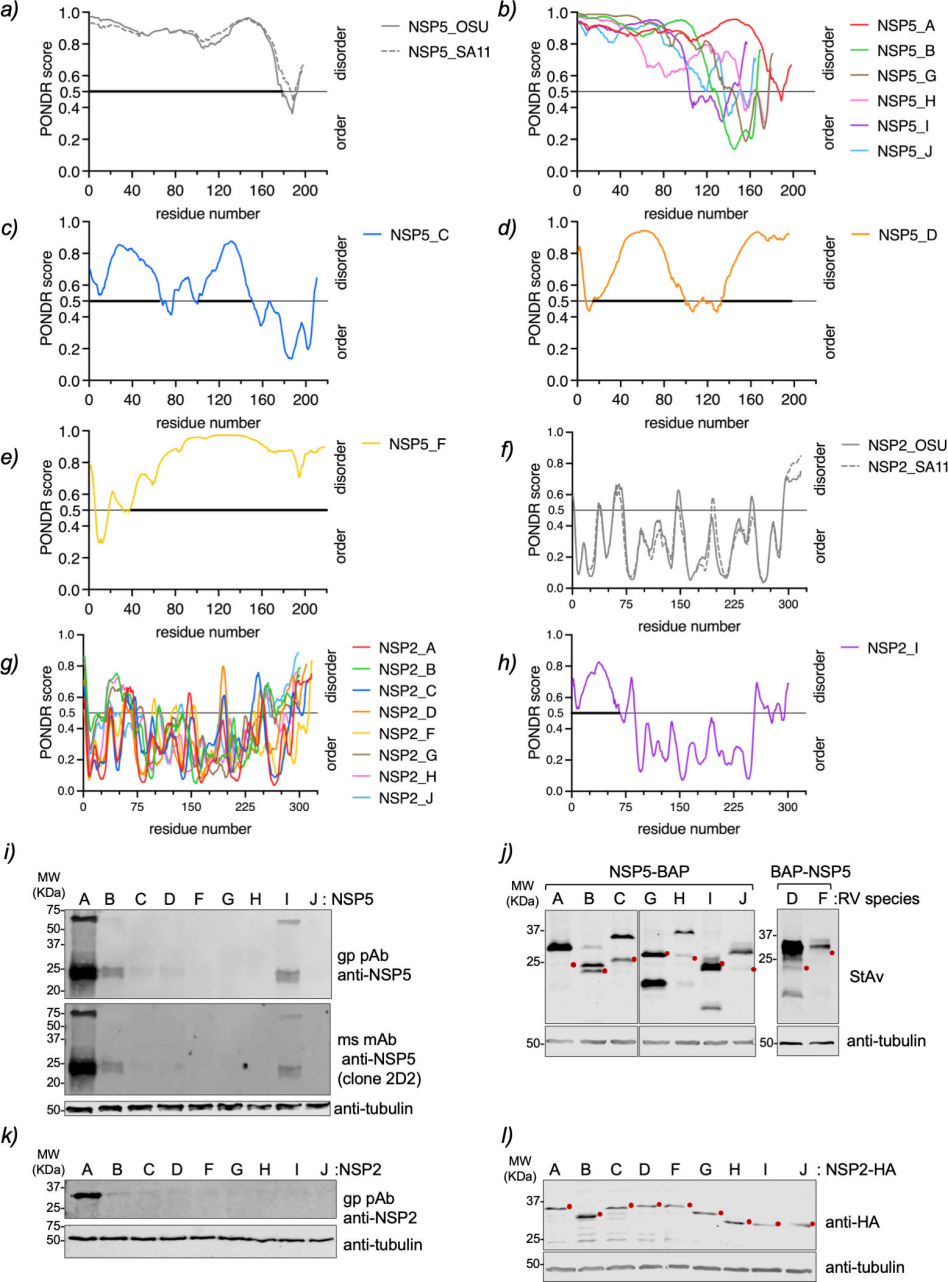
IDR and expression of NSP5 and NSP2 of species A to J. Plots comparing IDR prediction of NSP5 of RVA strains OSU and SA11 (a), RVA (strain SA11), RVB, RVG, RVH, RVI, and RVJ (b), RVC (c), RVD (d), and RVF (e). Plots comparing IDR prediction of NSP2 of RVA strains OSU and SA11 (f), RVA–RVH, RVG (g), and RVI (h). The bold part of the gray line highlights the disordered region in the protein. (i) Immunoblotting of MA104 cells expressing NSP5 of RVA to RVJ. The membrane was incubated with guinea pig polyclonal anti-NSP5 (top panel) and mouse monoclonal antibody (mAb) anti-NSP5 clone 2D2 (middle panel). (j) Immunoblotting of MA/cytBirA cells expressing NSP5-BAP of RVA-RVC, RVG-RVJ, and BAP-NSP5 of RVD and RVF. The membrane was incubated with streptavidin(StAv)-IRDye800. (k) Immunoblotting of MA104 cells expressing NSP2 of RVA–RVJ. The membrane was incubated with guinea pig polyclonal anti-NSP2. (l) Immunoblotting of MA104 cells expressing NSP2-HA of RVA to RVJ. The membrane was incubated with mouse mAb anti-HA. Anti-tubulin was used as a loading control. The red dot indicates the predicted molecular weight of the recombinant proteins.

Viroplasms, globular cytosolic replication compartments, have been described exclusively for RVA. No evidence points to the presence of these structures in other RV species. Interestingly, the co-expression of two RVA proteins, NSP5 and NSP2, can lead to the spontaneous formation of VLS, which are morphologically identical to viroplasms but unable to yield virus progeny. VLSs are excellent tools for studying the molecular details of the formation of viroplasms. In this context and in the absence of cell culture systems that allow investigating the replication of non-species A RVs, we assessed if homologous pairs of NSP5 and NSP2 of RV species B to J can form globular VLSs comparable to the ones observed in RVA. For this purpose, we identified in the NCBI data bank couples of NSP5 and NSP2 of RV species B to J (Table 1). As a reference for RVA, we used the open reading frames (ORFs) of NSP5 and NSP2 of simian strain SA11. The sequence alignment (Fig. S1) for NSP5 and NSP2 found poor similarity across the RV species, particularly for NSP5. The highest similarity for NSP5 RVA is found with RVF (45%, Table S1), and for NSP2 RVA, it is found with RVD (60.87%, Table S1). Even though these proteins have a similar number of residues, ranging from 157 (RVI) to 218 (RVF) for NSP5 and from 296 (RVG) to 318(RVF) for NSP2, they are quite diverse. Similar to NSP5 RVA (63), the NSP5 of the other RV species has a high content of serines and threonines, ranging from 14.37% for RVG to 23.74% for RVA (Table 1). NSP5 RVA has been demonstrated to be an intrinsically disordered protein (IDR) with an ordered C-terminal region of approximately 18 amino acids (30). In fact (Fig. 1a), this can be visualized using a PONDR score (http://www.pondr.com) that compares the primary amino acid sequences of NSP5 RVA from simian strain SA11 and porcine strain OSU. The PONDR score analysis showed the same disordered arrangement for most NSP5 RV species (Fig. 1b), except for NSP5 RVC, RVD, and RVF (Fig. 1c through e). Thus, NSP5 of RVC has central and C-terminal regions that are predicted to be ordered. Meanwhile, the NSP5 of RVD and RVF has a predicted ordered N-terminal region, with an additional central region ordered for the NSP5 of RVD. Consistent with the PONDR score, AlphaFold3 predicted an alpha helix at the C-terminal region of the monomeric NSP5 of RVA, RVB, RVG, RVH, RVI, and RVJ (Fig. S2a). Accordingly, an alpha helix is also predicted at the C-terminal region of NSP5 from RVC. However, the prediction of the presence of an alpha helix at either N- or C-terminal regions is of reduced confidence for RVD and RVF. Moreover, for RVD, an alpha helix is predicted in the NSP5 central region, between glutamic acid 73 and serine 111.

**TABLE 1:** NSP5 and NSP2 protein features of the RV species analyzed in this study

Rotavirus species	Host	Strain	NSP5				NSP2		
			GenBank accession number	Amino acid length	Predicted MW (kDa)	S/T (%)^*a*^	GenBank accession number	Amino acid length	Predicted MW (kDa)
RVA	Simian	SA11	BAW94621	198	21.72	23.74	BAW94618	317	36.56
RVB	Human	CAL-1	AF206724	170	19.77	16.47	AF205850	301	34.52
RVC	Porcine	12 R021	KP982878	210	23.21	19.05	KP982875	312	35.76
RVD	Chicken	05 V0049	NC_014521	195	22.26	16.93	NC_014518	310	35.88
RVF	Chicken	03 V0568	NC_021629	218	24.38	21.1	JQ920000	318	36.57
RVG	Chicken	03 V0567	JQ920012	181	20.84	14.37	JQ920009	300	34.5
RVH	Porcine	SP-VC36	MT644988	180	20.36	19.44	MT644956	296	33.29
RVI	Raccoon dog	SD-MO2	OM451078	157	17.72	22.29	OM451075	301	33.87
RVJ	Bat	BO4351	NC_055273	165	18.48	16.97	NC_055266	299	33.55

^*a*^ Percentage content of serines and threonines relative to the amino acid length.

In contrast, NSP2 of RV species A, B, C, D, F, G, H, and J has similar PONDR score patterns consistent with an ordered protein (Fig. 1f and g). The N-terminal region of NSP2 RVI is predicted to be disordered compared to NSP2 of the other RV species (Fig. 1h). NSP2 of RVA, RVB, and RVC has already been described as forming octamers consisting of two overlapping tetramers with a doughnut-shaped conformation (49, 64, 65). Interestingly, AlphaFold3 (66) predicts that NSP2 forms octamers with a doughnut-shaped conformation for all RVA species, A to J (Fig. S2b).

Each open reading frame of NSP5 and NSP2 of RV species B to J was chemically synthesized, cloned in expression plasmids, and tested for expression in MA104 cells by immunoblotting. As expected (Fig. 1i), staining with guinea pig anti-NSP5 (32, 33) and mouse monoclonal anti-NSP5 (clone 2D2) antibodies (67) resulted in strong NSP5/A but only weak NSP5/B and NSP5/I signals. Similarly (Fig. 1k), only NSP2/A was detected in immunoblotting with guinea pig anti-NSP2 antibody (48). These outcomes are consistent with the high antigenic diversity between NSP5 and NSP2 across RV species A to J. Therefore, NSP5 and NSP2 were fused to a sequence encoding a biotin acceptor peptide (BAP) and an influenza virus HA epitope as tags, respectively, to enable their detection through diverse biochemical assays. Specifically, NSP5/A, B, C, G, H, I, and J were fused at the C-terminus with a BAP tag (NSP5-BAP), and NSP5/D and F were fused at the N-terminus with a BAP tag (BAP-NSP5; Fig. 1j). All recombinant NSP5 proteins were shown to be correctly biotinylated when expressed in MA/cytBirA cells, a cell line expressing a cytosolic biotin ligase BirA (68), since they migrated at the predicted molecular weight (Table S2). The NSP2-HA proteins of the diverse RV species also proved to migrate at the expected molecular weights (Fig. 1l; Table S2).

### Characterization of VLS formation across RV species A to J

**Fig 2:**
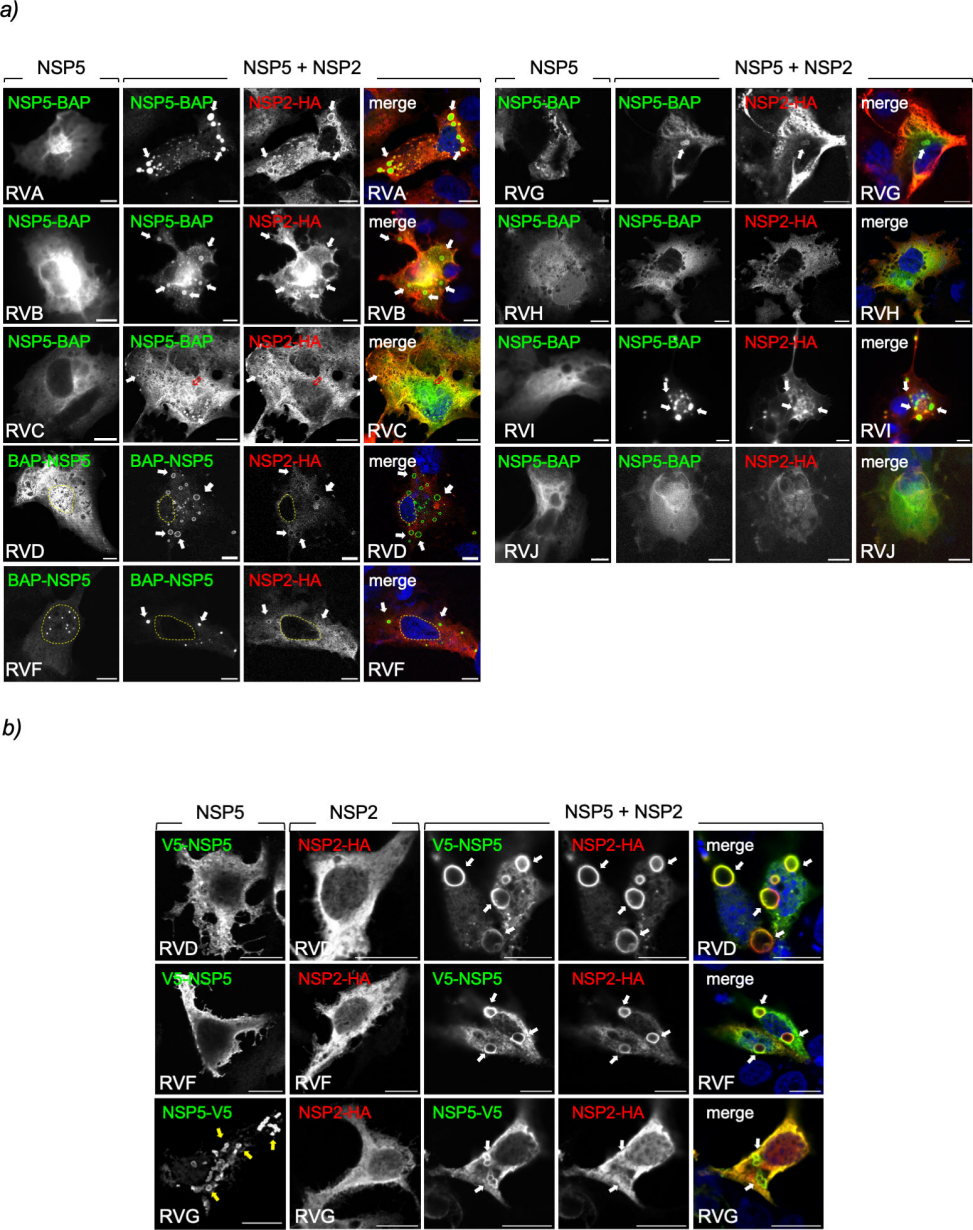
Characterization of VLS formation by co-expression of NSP5-BAP and NSP2-HA across RV species A to J. (a) Immunofluorescence images of MA/cytBirA cells expressing NSP5-BAP (RVA-RVC and RVG-RVJ) or BAP-NSP5 (RVD and RVF) alone (left column) or in co-expression with their respective NSP2-HA. At 16 hpt, the cells were fixed and immunostained for detection of NSP5-BAP (StAv, green, first and second columns) and NSP2-HA (anti-HA, red, third column). A merged image is shown in the right column of each panel. Nuclei were stained with DAPI (4′,6-diamidino-2-phenylindole) (blue). The scale bar is 10 µm. The white and open red arrows point to globular VLSs and filamentous structures, respectively. The discontinued yellow line labels the nucleus position as determined by DAPI staining. (b) Immunofluorescence images of LMH cells expressing V5-NSP5 (RVD and RVF) or NSP5-V5 (RVG; first column) or NSP2-HA (second column) individually or combined (third-fifth column). At 16 hpt, the cells were fixed and immunostained for detection of NSP5 (anti-V5, green) and NSP2 (anti-HA, red). A merged image is shown in the right column. The scale bar is 10 µm. The white arrows point to VLSs, and the yellow arrows point to the globular inclusion formed by NSP5-V5/G.

Next, we investigated if the co-expression of cognate NSP5 and NSP2 allowed the formation of VLS, which are defined by the colocalization of the signals of these two proteins in globular cytosolic inclusions. For this purpose, cognate couples of NSP5-BAP and NSP2-HA of RV species A to J were expressed in MA/cytBirA cells. As expected (Fig. 2a), the co-expression of NSP5-BAP with NSP2-HA of RVA produced VLSs (33). When inspecting the formation of globular cytosolic VLSs across RV species B to J, we found that RV species B, D, F, G, and I also formed globular inclusions upon co-expression of cognate NSP5-BAP with NSP2-HA. Interestingly, RVC formed a mixture of small globular inclusions and filamentous structures. We confirmed this result by co-expressing NSP5-BAP and NSP2-HA of RVC at diverse ratios (Fig. S3) and found that the overexpression of NSP5-BAP forms filamentous structures, while overexpression of NSP2-HA leads to the formation of globular VLSs. The filamentous structures dissolve upon nocodazole treatment, suggesting a dependence on the MT network. Moreover (Fig. S4), the co-expression of NSP5-BAP and NSP2-HA of RV species H and J did not lead to VLS formation at any of the tested pCI-NSP5-BAP:pCI-NSP2-HA transfection ratios (4:1, 2:1, 1:1, 1:2, and 1:4). NSP5-BAP and NSP2-HA of avian RV species D, F, and G behaved differently from the mammalian cohorts. In this context, BAP-NSP5/D appeared homogeneously distributed in the cytosol and nucleus, while BAP-NSP5/F forms globular nuclear inclusions, and NSP5-BAP/G forms aggregates in the cytosol. Interestingly, when BAP-NSP5/D, BAP-NSP5/F, or NSP5-BAP/G was co-expressed with their respective NSP2-HA, they were redistributed in the cytosol-forming VLSs. The numbers of RVG-VLSs were smaller than the ones observed for RVD and RVF VLSs. Adding a BAP tag at the C-terminus of NSP5/D or NSP5/F did not allow the formation of VLSs (data not shown). Because of the peculiar distribution pattern of avian NSP5 RV species D, F, and G, we investigated if the mammalian host environment provided by the MA104 cells could affect the distribution of these proteins. For this purpose (Fig. 2b), we expressed NSP5-V5 or NSP2-HA of RV species D, F, and G in chicken epithelial LMH cells. Of note, NSP5-V5 consists of an NSP5 ORF fused to a sequence encoding a simian virus V5 tag. Thus, V5-NSP5/D and F and NSP2-HA/D and F mainly localized homogeneously in the cytosol of the chicken cells. In contrast, NSP5-V5/G formed spontaneous globular cytosolic structures. The co-expression of NSP5-V5 and NSP2-HA of RV species D, F, and G in LMH cells led to the formation of globular cytosolic inclusions, particularly large for RVD and RVF. Interestingly (Fig. 2b), VLSs built-up of NSP5-V5 and NSP2-HA were apparently less abundant than globular cytosolic structures composed by NSP5-V5/G expressed alone.

### Role of NSP5 ordered region across RV species A to J

**Fig 3:**
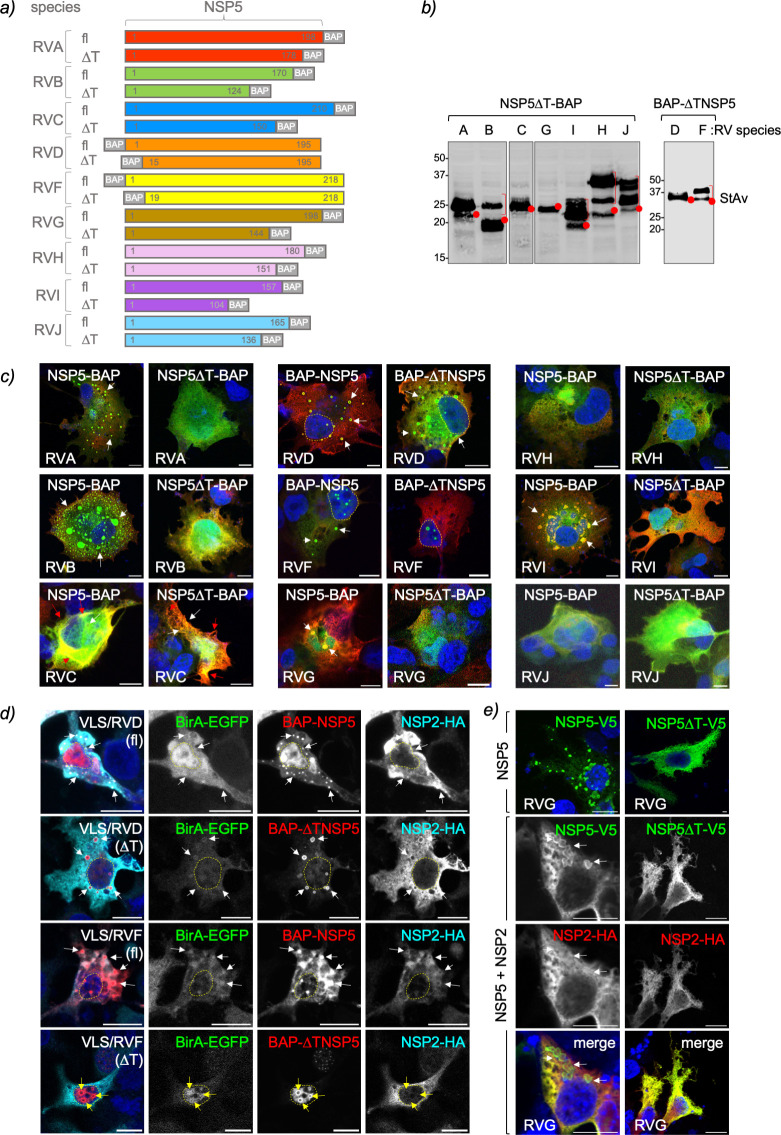
NSP5 ordered region, tail, is required for VLS formation among RV species. (a) Schematic representation of RV species A to J of full-length (fl) NSP5 and NSP5 with deleted tail region (∆T) fused to a BAP tag at the N- or C-terminus as indicated. b) Immunoblotting of MA/cytBirA cells expressing NSP5∆T-BAP (RVA to RVC and RVG to RVJ) and BAP-∆TNSP5 (RVD and RVF). The membrane was incubated with streptavidin-IRDye800. The red dots indicate the predicted molecular weight of the proteins. The red bracket shows a slow migration pattern of the protein. (c) Merged immunofluorescence images of MA/cytBirA cells co-expressing NSP2-HA with NSP5 or NSP5∆T fused to BAP tag. At 16 hpt, the cells were fixed and immunostained for the detection of NSP5 or NSP5∆T fused to BAP tag (StAv, green) or NSP2-HA (anti-HA, red). Nuclei were stained with DAPI (blue). The scale bar is 10 µm. (d) Immunofluorescence images of LMH cells co-expressing BirA-EGFP and NSP2-HA with BAP-NSP5 or BAP-∆TNSP5 of RVD and RVF. The cells were fixed at 16 hpt and immunostained for detection of BirA-EGFP (green, second column), NSP5 (StAv, red, third column), and NSP2 (anti-HA, cyan, fourth column). Nuclei were stained with DAPI (blue). The scale bar is 10 µm. A merged image is presented in the first column. (e) Immunofluorescence of LMH cells expressing NSP5-V5/G fl (NSP5-V5) or ∆T (NSP5∆T-V5) alone or together with NSP2-HA/G. After fixation, the cells were immunostained for the detection of NSP5 (anti-V5, green) and NSP2 (anti-HA, red). Nuclei were stained with DAPI (blue). The scale bar is 10 µm. A merged image for the co-expression of NSP5-V5 with NSP2-HA is shown. In (d) and (e), the white and red arrows point to globular and filamentous VLSs, respectively. The yellow arrows point to nuclear inclusions, and discontinued yellow lines label the nucleus position.

The NSP5 of RVA has an ordered region at its C-terminus corresponding to an alpha-helix (43, 69). It has been demonstrated that this region, ranging from amino acids 180–198 for simian strain SA11, termed tail, is necessary for NSP5 self-oligomerization (34, 45) and association with other RV proteins like NSP2, NSP6, VP2, and VP1 (34, 38, 40, 45, 70). A recombinant RV harboring NSP5 with a deleted tail region has an impaired replication because it cannot form viroplasms (37). Accordingly, we investigated if the ordered region of NSP5 in other RV species plays a role in VLS formation. For this purpose, we identified the ordered region in NSP5 from RV species A to J based on PONDR scores (Fig. 1b through e) and designed NSP5 deletion mutants lacking their tail region (∆T; Fig. 3a and b). For convenience, the ordered regions of NSP5/D and F, even if at the N-terminal region, are likewise denominated ∆T. The expression of the recombinant proteins was confirmed by immunoblotting (Fig. 3b). As expected (15, 32), the expression in MA/cytBirA cells of RVA NSP5∆T-BAP with NSP2-HA did not allow VLS formation (Fig. 3c). Similarly, the formation of globular VLS was impaired with NSP5∆T of the mammalian RV species B and I. The cytosolic distribution of NSP5∆T-BAP of RV species G, H, and J did not change in co-expression with their respective NSP2-HA, similar to the corresponding full-length NSP5-BAP. Interestingly, NSP5∆T-BAP/C shows a similar pattern as its full-length version when co-expressed with NSP2, forming mainly filamentous structures and a few punctate globular structures. Avian BAP-∆TNSP5/D formed VLS, while BAP-∆TNSP5/F formed distinct nuclear inclusions in MA/cytBirA cells that did not co-localize with NSP2-HA. Independently of the RV species tested, NSP2-HA remained homogeneously dispersed in the cytosol when in the presence of NSP5 with the deleted ordered region. A similar pattern of distribution of BAP-∆TNSP5 of RVD and RVF was observed for LMH cells (Fig. 3d). Thus, BAP-∆TNSP5/D forms VLSs, and BAP-∆TNSP5/F forms both cytosolic and nuclear inclusions when co-expressed with their respective NSP2-HA. NSP5∆T-V5/G expressed alone in LMH cells is homogeneously distributed in the cytosol, contrasting with full-length NSP5-V5/G, which forms cytosolic inclusions (Fig. 3e). Moreover, the co-expression of NSP5∆T-V5 and NSP2-HA of RVG had an impaired ability to form VLSs in LMH cells.

### NSP5 self-oligomerizes in RV species A to J

**Fig 4:**
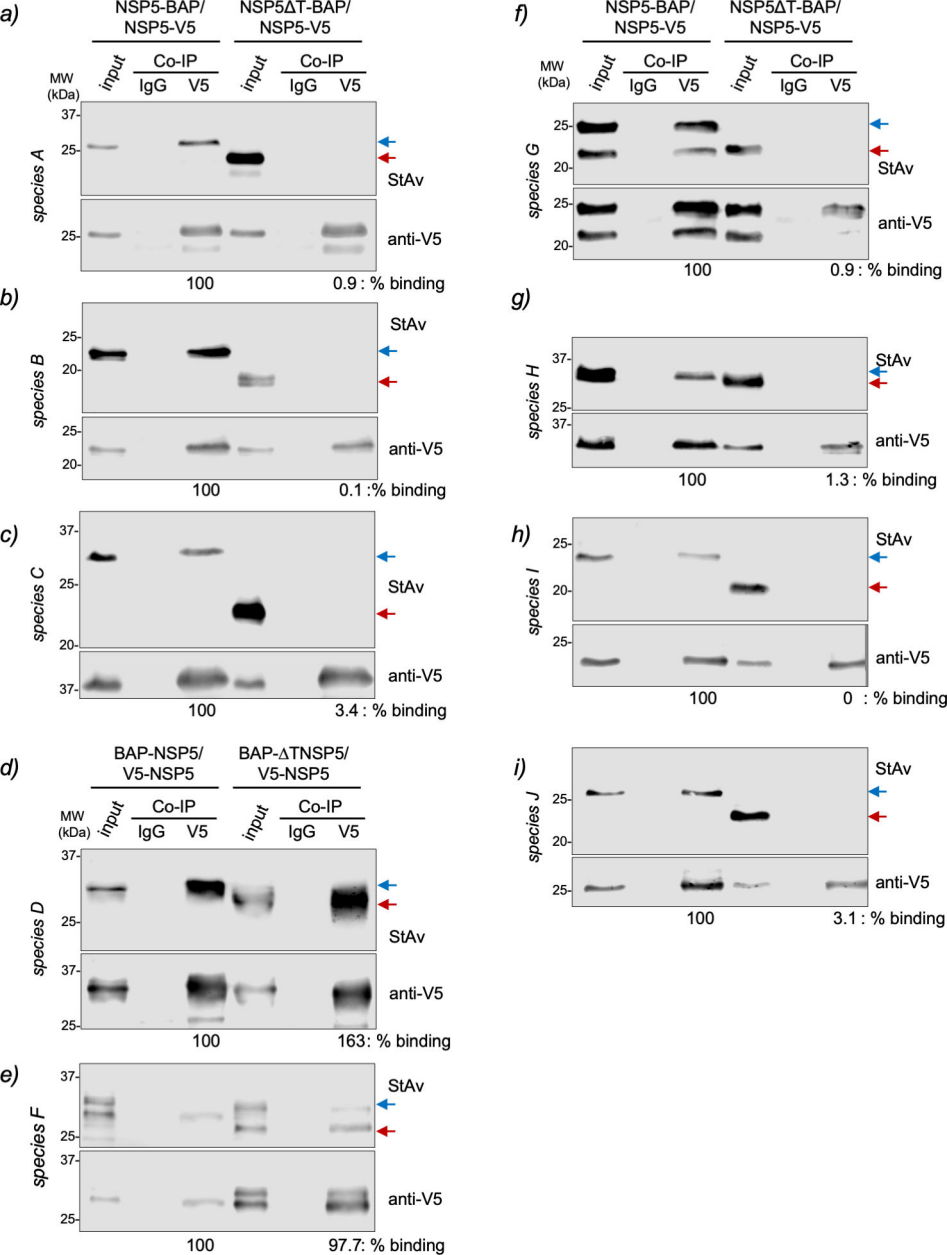
The NSP5 tail of RV species A to C and G to J is necessary for its self-oligomerization. Anti-V5 immunoprecipitated from extracts of MA/cytBirA cells co-expressing full-length NSP5-V5 with full-length NSP5-BAP or NSP5∆T-BAP for RV species A (a), B (b), C (c), G (f), H (g), I (h), and J (i). For RV species D (d) and F (e), the cells co-expressed full-length V5-NSP5 with full-length BAP-NSP5 or BAP-∆TNSP5. The membranes were incubated with streptavidin conjugated to IRDye800 (top panel) and mouse mAb anti-V5 (bottom panel). The input corresponds to 5% of crude cell extract. IgG corresponds to immunoprecipitation with isotype control antibody. Blue and red arrows point to full-length NSP5-BAP and NSP5∆T-BAP, respectively. The percentage of binding corresponds to the percentage of BAP-tagged NSP5 associated with V5-tagged NSP5.

A feature of RVA NSP5 corresponds to its ability to self-oligomerize (34, 45). Additionally, the C-terminal tail of NSP5 seems to be a requirement for VLS formation (37, 45). Therefore, we interrogated whether the tail region of NSP5 in the other RV species is also necessary for self-oligomerization. For this purpose, we added a V5 tag to the full-length NSP5 of all RV species (Fig. S5a and b) and co-expressed them with their respective BAP-tagged full-length NSP5 or NSP5∆T. Next, biotinylated cell extracts were immunoprecipitated with a monoclonal anti-V5 antibody to detect the association between NSP5-V5 and NSP5-BAP or NSP5∆T-BAP (Fig. 4). We found that NSP5 of all RV species tested could self-oligomerize, as denoted by the ability of NSP5-V5 to pull down full-length NSP5-BAP. However, NSP5-V5 was unable to pull down NSP5-BAP from RV species with tail deletion at the C-terminus (NSP5∆T-BAP), corresponding to RV species A, B, C, G, H, I, and J (Fig. 4a through c and f through i). The association of V5-NSP5 with BAP-NSP5∆T of RV species D and F (Fig. 4d and e) remained strong, suggesting no role of their N-terminal region for NSP5 self-oligomerization.

### NSP2 association with NSP5 across RV species A to J

**Fig 5:**
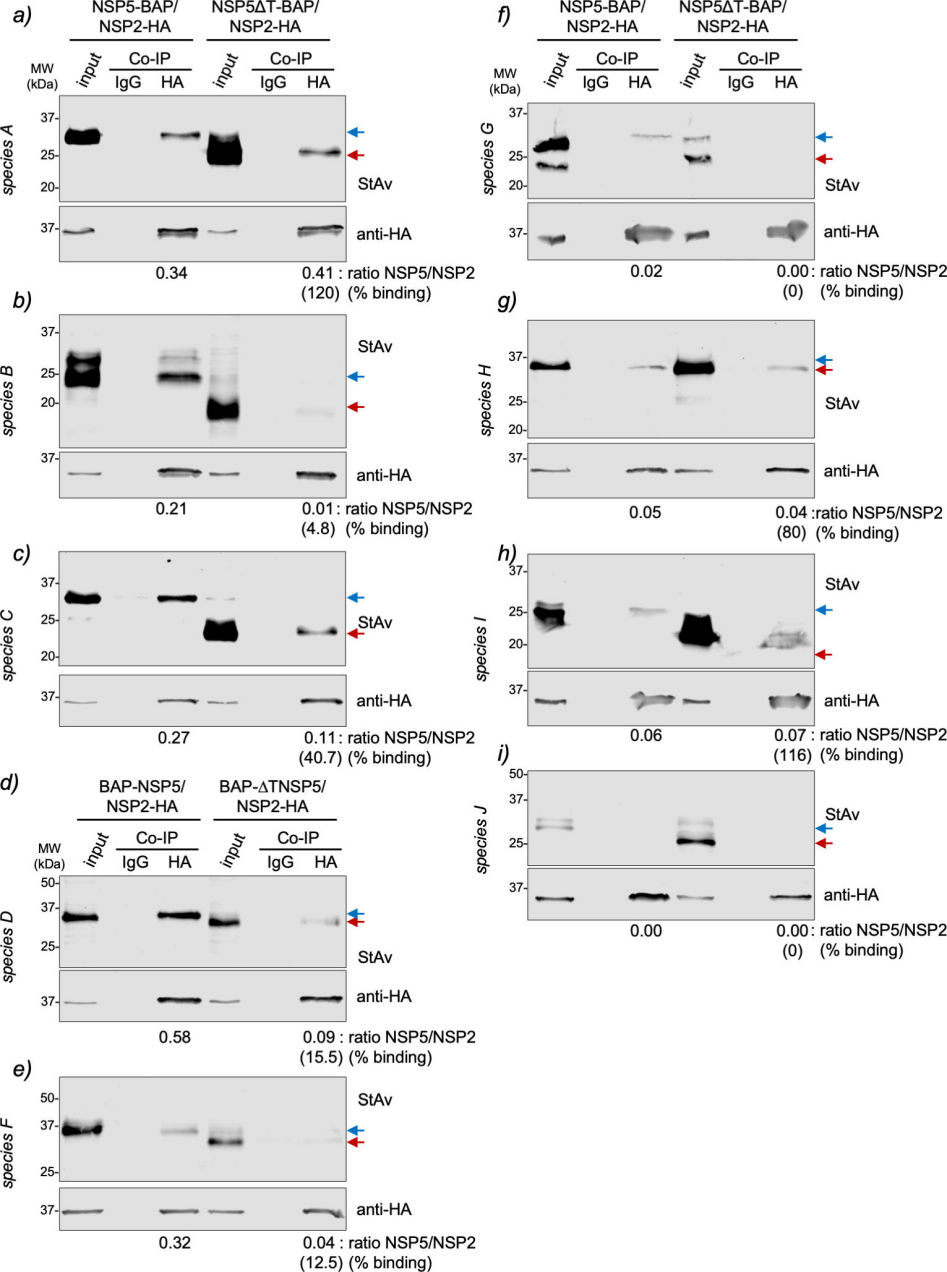
NSP5 requirements for association with NSP2 across RV species A to J. Anti-HA immunoprecipitated from extracts of MA/cytBirA cells co-expressing NSP2-HA with full-length NSP5-BAP or NSP5∆T-BAP for RV species A (a), B (b), C (c), G (f), H (g), I (h), and J (i). For RV species D (d) and F (e), the cells co-expressed NSP2-HA with full-length BAP-NSP5 or BAP-NSP5∆T. The membranes were incubated with streptavidin conjugated to IRDye800 (top panel) and mouse mAb anti-HA (bottom panel). The input corresponds to 5% of crude cell extract. IgG corresponds to immunoprecipitation with isotype control antibody. Blue and red arrows point to full-length NSP5-BAP and NSP5∆T-BAP, respectively. The NSP5/NSP2 association ratio of BAP-tagged full-length NSP5 and NSP5∆T to NSP2-HA is indicated. The percentage of binding of NSP5∆T to NSP2 relative to full-length NSP5 binding to NSP2 is indicated.

In RV species A, NSP2 associates with NSP5 by binding at the N-terminal region (amino acids 1–33) and its C-terminal tail (amino acids 180–198) (15, 70). In this context, we interrogated whether NSP2 binds to NSP5 in RV species B to J through their NSP5 tail region. For this purpose (Fig. 5), we co-expressed NSP2-HA with NSP5-BAP or NSP5∆T-BAP. The biotinylated cell extracts were immunoprecipitated with a monoclonal anti-HA antibody, followed by immunoblotting analysis of the pull-down complexes to detect BAP-tagged full-length NSP5 or NSP5∆T. As expected (Fig. 5a), NSP2 RVA binds to full-length NSP5-BAP but also to NSP5∆T-BAP (15). Except for NSP2/J (Fig. 5i), NSP2 of all RV species tested bind their full-length NSP5. Interestingly (Fig. 5b through f), NSP2-HA of RV species B, D, F, and G had a drastic impairment of the binding to BAP-tagged NSP5∆T, suggesting that NSP2 binds specifically to the predicted ordered regions of NSP5. Similar to NSP2-HA/A (Fig. 5g and h), the NSP2-HA of RV species C, H, and I do not show a reduction in the binding to NSP5 (depicted in Fig. 5 as the ratio of NSP5/NSP2 binding) when co-expressed with their respective NSP5∆T-BAP, suggesting that in these proteins, NSP2 binds to an additional region.

### Heterologous formation of VLS between RV species

Since some common elements are present among the diverse RV species, such as the ordered regions, the ability of NSP5 to oligomerize, and the capacity of some NSP2 species to form octameric structures, we assessed whether these common elements allow interspecies VLS formation. In the first instance (Fig. S6a; Table 2), we co-expressed NSP5/A with NSP2-HA of RV species A to J and checked for the formation of VLSs. As expected, homologous formation of VLS is observed for co-expression of NSP5 and NSP2-HA of RVA. Interestingly, NSP5/A forms globular VLS with NSP2-HA/I and filamentous and globular-like structures with NSP2-HA/C. By contrast, the combinations of NSP5/A with NSP2-HA of RV species B, D, F, G, H, and J did not lead to VLS formation, and the proteins remained homogeneously distributed in the cytosol of the cell. Next (Fig S6b; Table 2), we expressed NSP5-BAP of the RV species A to J with NSP2-HA/A. Only homologous pairs of NSP5-BAP with NSP2-HA of RVA formed VLSs in these specific conditions. Instead, the NSP5-BAP of the other RV species behaved as expressed without NSP2-HA (Fig. 2a).

**TABLE 2:** Formation of heterologous RV VLS

Co-expression^*a*^		VLS formation^*c*^	Co-expression^*b*^		VLS formation
NSP5 species	NSP2-HA species		NSP5-BAP species	NSP2-HA species	
RVA	RVA	+	RVA	RVA	+
RVA	RVB	−	RVB	RVA	−
RVA	RVC	+, filamentous/globular	RVC	RVA	−
RVA	RVD	_	RVD	RVA	−, nuclear aggregates
RVA	RVF	−	RVF	RVA	−, nuclear inclusions
RVA	RVG	−	RVG	RVA	−, aggregates
RVA	RVH	−	RVH	RVA	−
RVA	RVI	+	RVI	RVA	−
RVA	RVJ	−	RVJ	RVA	−

^*a*^ Confirmed by co-immunostaining of NSP5 of RVA and NSP2 HA of species A–J using specific guinea pig pAb anti-NSP5 and mAb anti-HA, respectively.

^*b*^ Confirmed by co-immunostaining of NSP5-BAP of species A to J and NSP2-HA of RV species A using StAv and mAb anti-HA, respectively.

^*c*^ The result corresponds to the observation of three independent experiments.

**Fig 6:**
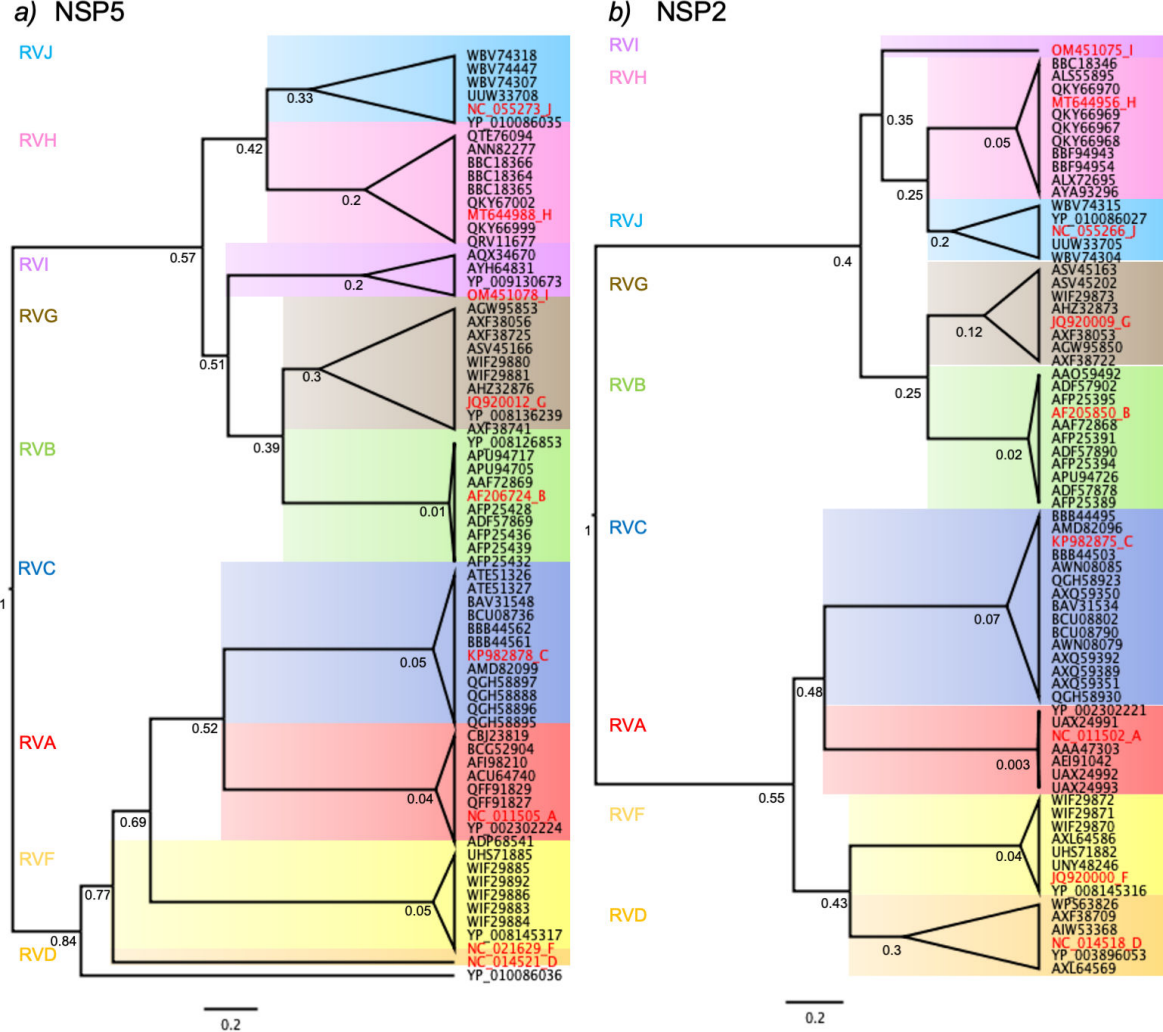
Phylogenetic trees for amino acid sequences of NSP5 and NSP2 in RV species A to J. Maximum likelihood tree showing phylogenetic relationships between NSP5 (a) and NSP2 (b) genes of RV species A to J. The red label corresponds to the cCDS used in this study, followed by a letter associated with its RV species. Each RV species has a colored panel: RVA, red; RVB, green; RVC, blue; RVD, orange; RVF, yellow; RVG, brown; RVH, pink; RVI, violet; and RVJ, light blue. The bootstraps were determined as the node ages with a time scale root set to 1. The scale is 0.2 substitutions per nucleotide.

**Fig 7:**
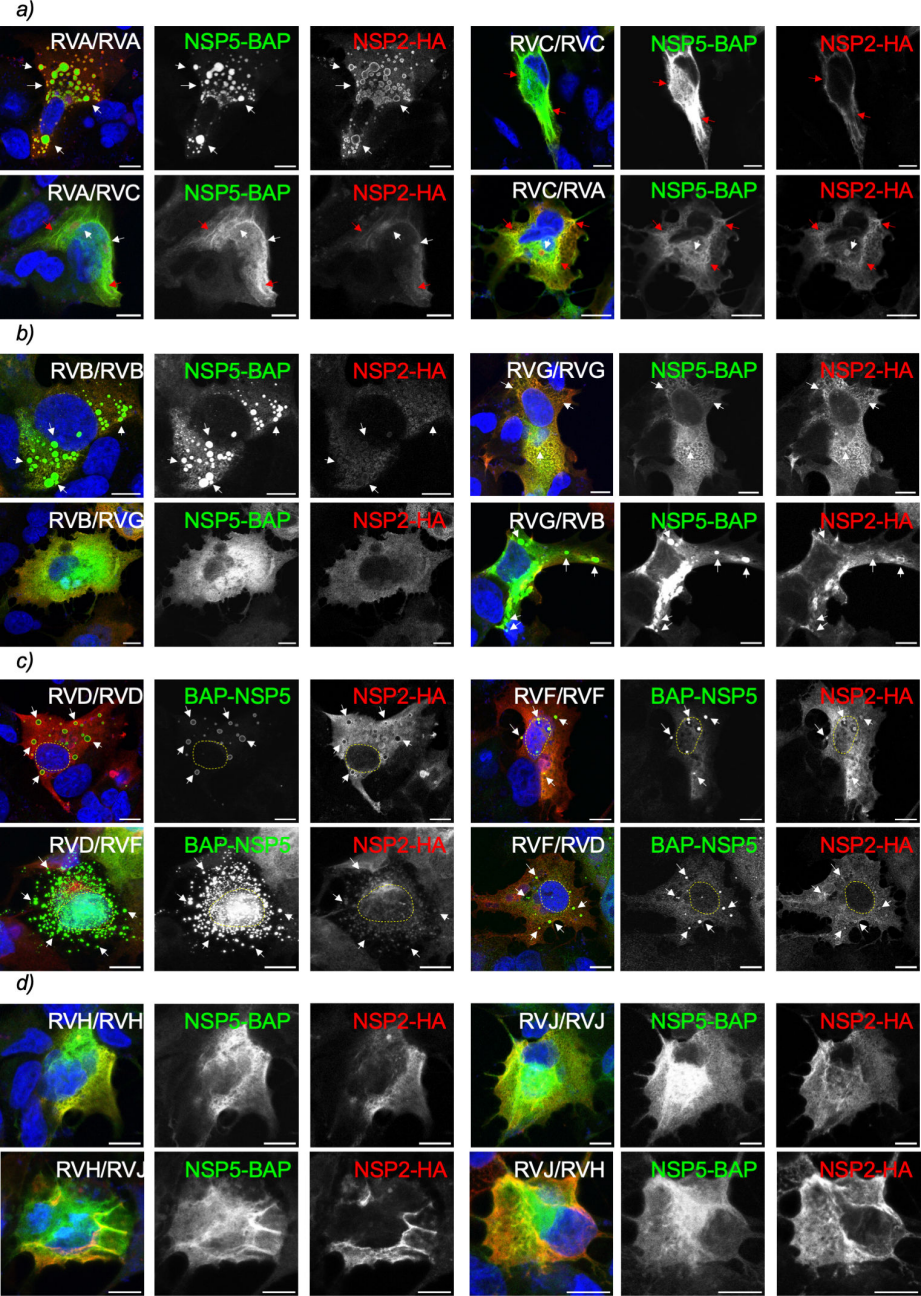
Heterologous formation of VLS among closely related RV species. Immunofluorescence images of MA/cytBirA cells co-expressing NSP5 tagged to BAP and NSP2-HA of closely related RV species A and C (a), B and G (b), D and F (c), and H and J (d). After fixation, the cells were immunostained for the detection of NSP5 (StAv, green) and NSP2 (anti-HA, red). The nuclei were stained with DAPI (blue). The indication at the top right corner corresponds to the RV species of NSP5 and NSP2, respectively. The scale bar is 10 µm. The white and red arrows point to globular and filamentous VLSs, respectively. The yellow lines label the nucleus position.

Next, we compared the evolutionary proximity of NSP5 and NSP2 of the diverse RV species (Fig. 6) based on their nucleotide coding sequences (CDS) available in the data bank to form pairs of RV species with common ancestors. Interestingly, the phylogenetic trees of NSP5 and NSP2 present similar topologies for the analyzed RV species. Thus, RVA is closer to RVC, RVB is closer to RVG, RVD is closer to RVF, RVH is closer to RVJ, and RVI shares a common ancestor with RVB, RVG, RVJ, and RVH for both protein-coding sequences. Consistent with this finding, some AlphaFold3-predicted NSP5 monomers showed structural similarities among them, such as RVH, RVI, and RVJ or RVB and RVG. In this context (Fig. 7a), we assessed VLS formation by co-expressing NSP5-BAP and NSP2-HA of RV species A and C in all four interspecies combinations of NSP2 and NSP5 (A/A, C/C, A/C, and C/A). Only NSP5-BAP/A with NSP2-HA/A formed globular cytosolic VLS, while the other combinations formed a mixed population of filamentous and globular VLSs. The combinations of NSP5-BAP and NSP2-HA of RVB and RVG (Fig. 7b) showed VLS formation by co-expression of NSP5-BAP/B or NSP5-BAP/G with NSP2-HA/G. Interestingly (Fig. 7c), avian BAP-NSP5 and NSP2-HA of RVD and RVF formed VLS in all the combinations. As expected (Fig. 7d), the combinations of NSP5-BAP with NSP2-HA of RV species H and J did not lead to globular VLS formation.

### Chimeric NSP5∆T/H and J with tail of NSP5/A form VLS

**Fig 8:**
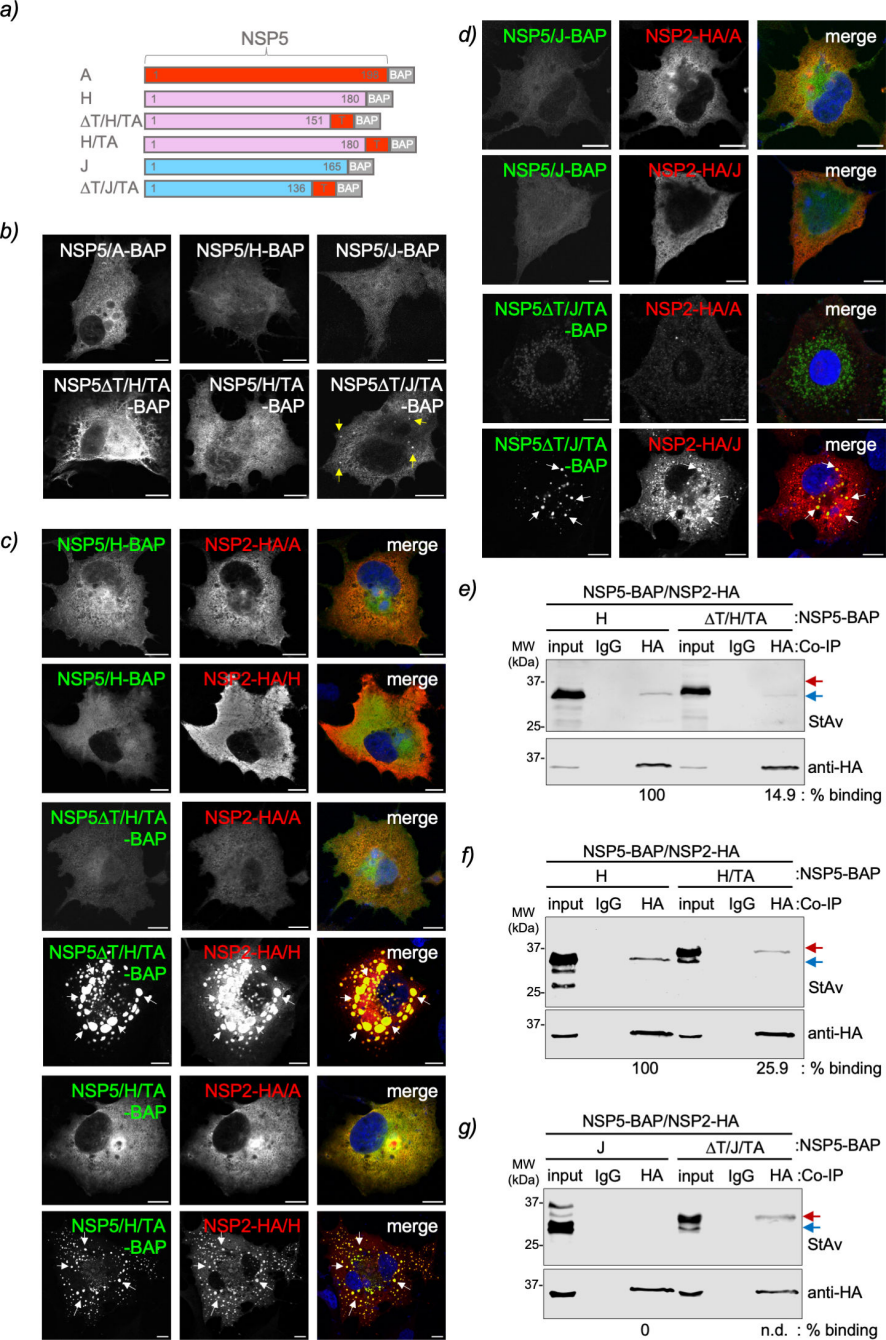
Chimeric NSP5 RVH and RVJ harboring RVA tail form VLS. (a) Schematic representation of chimeric NSP5 RVH and RVJ with the NSP5 RVA (TA) tail fused to a BAP tag at the C-terminal region. (b) Immunofluorescence images of MA/cytBirA cells expressing wild-type NSP5 and chimeric NSP5 of RVH and RVJ. The yellow arrows point to globular cytosolic inclusions. (c) Immunofluorescence images of MA/cytBirA cells co-expressing NSP5/H-BAP (top panel), NSP5∆T/H/TA-BAP (middle panel), and NSP5/H/TA (bottom panel) with either NSP2-HA/A or NSP2-HA/H. (d) Immunofluorescence images of MA/cytBirA cells co-expressing NSP5/J-BAP (top panel) and NSP5∆T/J/TA-BAP (bottom panel) with either NSP2-HA/A or NSP2-HA/J. The cells were fixed at 16 hpt and immunostained for the detection of NSP5 (StAv, green) and NSP2 (anti-HA, red). The nuclei were stained with DAPI (blue). The scale bar is 10 µm. The white arrows point to globular VLSs. Anti-HA immunoprecipitated from extracts of MA/cytBirA cell co-expressing NSP2-HA/H with NSP5∆T/H/TA (e) or NSP5-H/TA (f) and co-expressing NSP2-HA/J with NSP5∆T/J/TA (g). As a control, wild-type NSP5 for each tested RV species is included. The membranes were incubated with streptavidin conjugated to IRDye800 (top panel) and mouse mAb anti-HA (bottom panel). The input corresponds to 5% of crude cell extract. IgG corresponds to immunoprecipitation with isotype control antibody. Blue and red arrows point to full-length NSP5-BAP and NSP5∆T-BAP, respectively. The percentage of binding of BAP-tagged full-length NSP5 and NSP5∆T to NSP2-HA is indicated.

Our results demonstrated that NSP5/H and J could not form VLS with their respective NSP2, even though their tail regions appear necessary for oligomerization (Fig. 4g and i). However, the tail regions alone seem insufficient for a strong interaction with NSP2 (Fig. 5g and i). Additionally, we have shown that NSP5 and NSP2 from certain RV species can be swapped (Fig. 7), which allows for VLS formation. In addition, it has been shown that the short-tail region of NSP5/A (amino acids 180–198) is essential for viroplasm formation (37). We next investigated whether replacing the predicted ordered region of NSP5/H and NSP5/J with the tail of NSP5 (TA) would enable the VLS formation with NSP2 of their respective species. For this purpose (Fig. 8a; Fig. S4c and d), we built two chimeric proteins harboring NSP5∆T/H or NSP5∆T/J fused to the TA followed by a BAP-tag, termed NSP5∆T/H/TA-BAP and NSP5∆T/J/TA-BAP, respectively. Additionally, we built a third chimeric protein composed of full-length NSP5/H fused to TA (NSP5/H/TA-BAP). Interestingly (Fig. 8b), NSP5∆T/H/TA-BAP and NSP5/H/TA-BAP expressed alone were found to be homogeneously dispersed in the cytosol of the cells, whereas NSP5∆T/J/TA formed discrete cytosolic inclusion resembling VLSs. As expected, VLSs are not formed by the co-expression of NSP5-BAP/H with either NSP2-HA/A or NSP2-HA/H (Fig. 8c, top panel), the co-expression of NSP5-BAP/J with either NSP2-HA/A or NSP2-HA/J (Fig. 8d, top panel), or the co-expression of NSP2-HA/A with either NSP5∆T/H/TA-BAP or NSP5∆T/J/TA-BAP (Fig. 8c and d, top-middle panels). By contrast, the co-expression of chimeric NSP5∆T/H/TA-BAP with NSP2-HA/H (Fig. 8c, middle panel) or NSP5∆T/J/TA-BAP with NSP2-HA/J (Fig. 8d, bottom panel) resulted in well-discernible globular VLSs. Moreover, adding TA to the full-length NSP5/H when co-expressed with NSP2-HA/H also allowed the formation of VLSs (Fig. 8c, bottom panel). Using an immunoprecipitation assay, we then tested the ability of these chimeric proteins to associate with their respective NSP2s. Our results demonstrated that NSP2-HA/H binds to BAP-tagged NSP5/H, NSP5∆T/H/TA, and NSP5/H/TA (Fig. 8e and f). However, this binding was apparently weaker for NSP5/H/TA-BAP and NSP5∆T/H/TA-BAP than for NSP5-BAP/H. Interestingly (Fig. 8g), the chimeric NSP5∆T/J/TA-BAP can associate with NSP2-HA/J, contrasting the wild-type NSP5-BAP/J, which cannot bind to NSP2-HA/J.

## DISCUSSION

A recurrent question in the rotavirus research field relates to the formation of viroplasms in non-RVA species. The accepted ICTV RV species A to J share common features, such as 11 dsRNA genome segments with conserved 5'- and 3'-terminal untranslated sequences. Moreover, the open reading frames on these genome segments encode proteins with primary amino acid sequences homologous to RVA species, including the proteins involved in viroplasm formation (NSP5, NSP2, and VP2) and replication (VP1, VP2, VP3, and VP6). Even if all the elements facilitating the formation of viroplasms are present, it remains challenging to demonstrate their existence due to the lack of tools for viroplasm recognition. These include the scarcity of specific antibodies directed against proteins of every RV species and the lack of cell culture systems that support the replication of non-RVA species, making it difficult to identify viroplasms using electron microscopy techniques. In this study, we extrapolated the knowledge acquired from RVA viroplasms and used the co-expression of NSP5 and NSP2 of the RV species B to J to detect the formation of globular VLSs. These structures, even if unable to replicate, have been demonstrated to be simplified models for studying highly complex viroplasms in RVA (15, 16, 31, 33, 40, 51, 71), allowing even the investigation of the recruitment of cellular proteins as was recently demonstrated for the TRiC chaperonin (68). As summarized in Table 3, our experiments revealed that co-expressed NSP5 and NSP2 of species B, D, F, G, and I form VLSs that are morphologically similar to those observed for RVA. We found that only some of the RV species, specifically C, H, and J, were unable to form globular VLS upon co-expression of NSP5 and NSP2. For NSP5 and NSP2 of RVC, we observed a mixed population of globular and filamentous structures that depended on the expression levels of the two proteins, shifting toward globular structures when NSP2 is over-expressed. In the standard conditions for detecting VLS formation, the RVC pair formed nocodazole-sensitive filamentous structures, suggesting direct dependence on the MT network. It is not surprising to observe filamentous VLS since viral factories of some members of the *Reoviridae* family also form filamentous inclusion bodies. For example, the µ2 protein of mammalian orthoreovirus (MRV) strain T1L, which can bind to MTs, forms filamentous viral factories, while the µ2 protein of strain T3D^N^, which cannot bind to MTs, forms globular viral factories (72). Additionally, we found that VLS formation in avian RV species D, F, and G is host-dependent since these structures formed better in avian LMH cells than in mammalian MA104 cells. It is particularly true for RVG, where NSP5 expressed alone forms aggregates in MA104 cells, while in LMH cells, it forms regularly shaped globular inclusions. NSP5 of RVF represents another interesting case since it forms globular nuclear inclusions when expressed alone in MA104 cells but not in LMH cells. Of note, NSP5 has an average molecular weight below 30 kDa, which enables its passive diffusion to the nucleus (73) when expressed independently of other RV proteins. This suggests that RV proteins like NSP2 may play an accessory role in retaining NSP5 in the cytosol. We infer from our results that in RVF, NSP2 retains NSP5 in the cytosol. However, we cannot rule out the possibility that NSP5 from all RV species transiently moves to the nucleus through active nuclear transport. The ability of NSP5 from RVF and RVG to form inclusions is novel in the context of the RV field. However, this phenomenon is not unique within the *Reoviridae* family; for instance, the MRV protein µNS forms cytosolic inclusions when expressed in the absence of other viral proteins (74, 75). The NSP5/NSP2 pairs of RVH and RVJ do not lead to VLS formation at any of the tested ratios, indicating that either these two proteins are not sufficient to form VLS and require additional RV proteins like VP2 (31, 40) or that viroplasms in these species are composed of other RV proteins not related to NSP5 or NSP2.

**TABLE 3:** Summary of structural NSP5 requirements for known replication functions across RV species A to J

RV species	Globular VLS formation^*a*^		NSP5 oligomerization^*b*^		NSP5 interaction with NSP2^*c*^	
	fl NSP5^*g*^	NSP5∆T	fl NSP5	NSP5∆T	fl NSP5	NSP5∆T
A	+	−	+	−	+	+
B	+	−	+	−	+	±
C	−^*d*^	−	+	−	+	+
D	+	+	+	+	+	±
F	+	+^*e*^	+	+	+	±
G	+^*f*^	−	+	−	±	−
H	−	−	+	−	±	±
I	+	−	+	−	±	±
J	−	−	+	−	−	−

^*a*^ Confirmed by co-immunostaining for the detection of both VLS components, NSP5 and NSP2 (transfection ratio 1:2 = NSP5:NSP2).

^*b*^ Confirmed by co-immunoprecipitation of NSP5-V5 using anti-V5 antibody.

^*c*^ Confirmed by co-immunoprecipitation of NSP2-HA using anti-HA antibody. Weak association determined by an NSP5/NSP2 ratio below 0.1.

^*d*^ Form filamentous cytosolic structures. Upon overexpression of NSP2 (ratio 1:8 = NSP5:NSP2), globular VLS can be detected.

^*e*^ Forms nuclear inclusions.

^*f*^ Forms VLS only in chicken epithelial LMH cells.

^*g*^ +, positive; ±, weak; −, negative.

Interestingly, the NSP5 proteins of all the RV species tested in this study were predicted to be intrinsically disordered, similar to NSP5 of RV species A. In this context, it is known that NSP5/A can self-oligomerize, a feature that is conserved in NSP5 of species A to J, as demonstrated by co-immunoprecipitation assays. The self-oligomerization of NSP5 in RVA through its tail is essential for viroplasm formation (15, 37). Thus, recombinant RVA NSP5 with a tail deletion does neither form viroplasms nor replicate if not rescued by a cell line expressing full-length NSP5 (37). Additionally, the tail of NSP5 is required to associate with other virus proteins such as NSP2 (15), VP1 (38), VP2 (40), or NSP6 (34). In this context, the deletion of the ordered region at the C-terminal end (RV species A, B, G, and I) in NSP5 hampered self-oligomerization, which is directly correlated with their ability to form globular VLSs. However, deleting the predicted ordered NSP5 N-terminal region of RV species D and F did not affect oligomerization or the ability to form globular inclusions. Interestingly, the deletion of the N-terminus of NSP5/F led to the formation of nuclear inclusions even in co-expression with NSP2. These results suggest that the binding site to NSP2 is localized at its N-terminal region, that NSP2 retains NSP5/F in the cytosol, and that the ability to form inclusions depends on the center or C-terminal region of NSP5/F. Consistent with our results, AlphaFold3 predicts (Fig. S7) that the full-length NSP5 dimerizes through its intertwined C-terminal tail in RV species A, B, C, G, H, I, and J. The deletion of this tail appears to disrupt the intertwined interaction between the NSP5 monomeric structures, hampering their oligomerization. However, the intertwined tail between NSP5/D and F is not observed by AlphaFold3 at either N- or C-terminal regions, suggesting that these proteins oligomerize through another undetermined region or mechanism.

Based on PONDR-score predictions, we chose to tag NSP5 at the ordered region, which was at the C-terminus for RV species A, B, C, G, H, I, and J, and at the N-terminus for RV species D and F. This is consistent with previous studies that showed NSP5 of RVA tagged at the C-terminus was able to form VLS with either NSP2 or VP2 (15, 16, 24, 30, 31). In contrast, if NSP5 of RVA was tagged at its N-terminal region, it formed spontaneous inclusions (76, 77). In addition to NSP5 of RVA, we observed that when tagged at the C-terminus, NSP5 of RV species B, C, G, and I formed VLS with their cognate NSP2. Interestingly, in RVD and RVF, tagging the N-terminus of NSP5 supported the formation of VLS with the respective NSP2, while tagging the C-terminus did not (data not shown). Thus, it appears that the ability of a tagged NSP5 to form VLS with NSP2 correlates with the predicted ordered region found at the C-terminus or N-terminus. Similarly, NSP2 across RV species A to J was tagged at the C-terminus according to previous reports (15, 30, 65) and its resemblance to their PONDR prediction pattern.

In RVA, NSP2 associates with NSP5 through its N-terminus (amino acid region 1–33) and tail (amino acid region 190–198) (15, 70). These results are consistent with our NSP2-HA/NSP5-BAP-binding assay for RVA pairs, as the deletion of the NSP5 tail still allows association with NSP2. This suggests the presence of an additional binding site for NSP2, potentially corresponding to the NSP5 N-terminal region. The biochemical properties of NSP5 and NSP2, such as their continuous nucleotide transfer promoting phosphorylation, the NTPase activity of NSP2, and the high peptide chain flexibility of NSP5 (43, 50), require their association to be stabilized by chemical crosslinking for detection. Similarly, the NSP2 and NSP5 association must be chemically stabilized in RV species B to I for detection. However, the association of NSP5 to NSP2 seems eight times stronger in RV species A, B, C, D, and F than in RV species H, G, and I, as denoted by the estimation of their relative binding ratios. Interestingly, NSP2/J does not associate with NSP5 at any of the tested conditions of the DSP [dithio(succinimidyl propionate)] crosslinker. As described previously using crystallography and cryo-electron microscopy, NSP2 of RV species A (49), B (64), and C (65) has octameric structures with doughnut-shaped disposition. Consistent with these observations, AlphaFold3 also predicted that NSP2 of RV species A to J is organized as octamers (Fig. S2b). These outcomes suggest that NSP5 is responsible for orchestrating the association with NSP2, where NSP5 initially oligomerizes and then associates with NSP2. It is noticed that a positive association of NSP5 and NSP2 does not necessarily lead to globular VLS formation, for example, for the pair of RVC (Table 3). As expected, the deletion of the NSP5 tail does not disrupt the association with NSP2, for example, for RVA, RVC, RVH, and RVI, probably due to the association with another region of NSP5. However, the association was drastically hampered in RV species D, F, and G when the ordered region of NSP5 was deleted.

An interesting novel observation relates to the interspecies formation of NSP5 and NSP2 VLSs. We discovered that closely related RV species can exchange these proteins to form globular VLS. The RV species B and G, along with the avian RV species D and F, have been shown to possess promiscuous NSP5 and NSP2 proteins capable of forming interspecies globular VLSs. As a result, they can form globular VLS with any combination of NSP5 and NSP2. This result suggests that, at least in avian RVs, interspecies reassortment of the viroplasm-forming proteins is possible. The use of chimeric NSP5/H and J, having incorporated the tail of NSP5/A, allowed the formation of VLS exclusively with their cognate NSP2 but not with NSP2-HA/A. This indicates that cognate couples of NSP2 and NSP5 associate specifically in a region independent of their NSP5 tail. Moreover, the NSP5 tail A stabilizes these associations, suggesting that other RV proteins could favor RV species H and J in forming viroplasms. It is well known that VP2 also allows the formation of VLS (31, 40). Nevertheless, VP6 also improves the stabilization of viroplasms, at least in RV species A (71). In this context and based on the data reported, we cannot exclude that post-translational modifications, such as specific phosphorylation, O-glycosylation, or sumoylation of both NSP5 and NSP2, could also influence the formation of VLS and viroplasms in non-RVA species. The non-canonical site for casein kinase 1-alpha (CK1-alpha; Fig. S1a) that is primed in NSP5 serine 67 of RVA strain SA11 is conserved in RV species A, C, D, and F. Surprisingly, the closely related RV species B and G have replaced the serine 67 by a tyrosine, which may be phosphorylated by CK1-alpha. RV species H, I, and J have obliterated this phosphorylation site. Coincidentally, the NSP2 phosphorylation site for CK1-alpha in RVA corresponding to serine 313 is also conserved in RV species A, C, D, and F (Fig. S1a). Additional studies need to be performed to prove the role of these phosphorylation sites in VLS and viroplasm formation across the RV species.

The findings of this study could potentially expand the applicability of the RV reverse genetics technology for other RV species. The current plasmid-only-based reverse genetics system for RV has been limited to certain strains of RVA (60–62, 78, 79). A significant advancement in RVA reverse genetics technique was achieved through the co-transfection of plasmids encoding NSP5 and NSP2 ORFs along with plasmids encoding the 11 genome segments (62). This method relies on NSP5 and NSP2 forming VLSs to shield newly generated virus RNA from the host innate immune system. It should be noted, however, that certain NSP5/NSP2 pairs from non-RVA species, such as RVH and RVJ, did not support VLS formation, while others, like RVC pairs, exhibited globular and filamentous patterns.

In conclusion, when co-expressed with NSP2, NSP5 from certain RV species forms VLSs that resemble RVA viroplasms. These specific NSP5 proteins, unlike those that do not support VLS formation, self-oligomerize primarily through a predicted ordered region located mainly at the C-terminus. This study paves the way for understanding the pathophysiology of various RV species.

## MATERIALS AND METHODS

### Cells and viruses

MA104 cells (embryonic rhesus monkey kidney, ATCCCRL-2378, RRID: CVCL_3845) were cultured in Dulbecco’s modified Eagle’s medium (DMEM, GibcoBRL) supplemented with 10% fetal calf serum (FCS; AMIMED, BioConcept, Switzerland) and penicillin (100 U/mL)-streptomycin (100 µg/mL; Gibco, Life Technologies). MA/cytBirA (24) cell lines were grown in DMEM supplemented with 10% FCS, penicillin (100 U/mL)-streptomycin (100 µg/mL), and 5 µg/mL puromycin (InvivoGen, France). LMH cells (chicken hepatocellular carcinoma epithelial, ATCCCRL2117) were cultured in Waymouth’s MB752/1 (Sartorius) medium supplemented with 10% FCS and penicillin (100 U/mL)-streptomycin (100 µg/mL).

The recombinant vaccinia virus encoding T_7_ RNA polymerase (strain vvT7.3) was amplified as previously described (80).

### Antibodies and reagents

Guinea pig anti-NSP5, mouse monoclonal (mAb) anti-NSP5 (clone 2D2), and guinea pig anti-NSP2 were described previously (15, 67). Mouse mAb anti-HA (clone HA-7) and mouse anti-tubulin (clone B5-1-12) were purchased from Merck. Mouse mAb anti-V5 (SV5-PK1) was purchased from Abcam. Mouse MAb anti-α-tubulin was directly conjugated to Atto 488 using the Lightning-Link Atto 488 conjugation kit from Innova Bioscience, United Kingdom. Alexa Fluor594 anti-HA.11 (clone 16B12) was purchased from BioLegend. Streptavidin-DyLight 488 and mouse secondary antibodies conjugated to Alexa 488 or Alexa 594 were purchased from Thermo Fisher Scientific. Streptavidin-IRDye800CW and secondary antibodies for immunoblot conjugated to IRDye680 and IRDye800CW were purchased from LI-COR.

Nocodazole was purchased from Sigma. Dithio(succinimidyl propionate) was purchased from ThermoFisher Scientific.

### Rotavirus sequences

The rotavirus NSP5 and NSP2 open reading frames from species B to J were synthesized at GenScript using the sequences described in the supplemental material. NSP5 B to J was cloned in pCI-Neo (Promega) between *Mlu*I/*Not*I restriction sites. NSP2 B to J was cloned in pCI-Neo (Promega) between *EcoR*I/*Not*I restriction sites. The GenBank accession number of sequences for NSP5 and NSP2 used in this study is summarized in Table 1.

### Plasmid constructs

pCI-NSP5-BAP/A, B, C, G, H, I, and J were obtained by PCR amplification of pCI-NSP5/A, B, C, G, H, I, and J using specific primers to insert *Mlu*I and BAP tag/*Not*I sites, followed by ligation into those sites in pCI-Neo (Promega). pCI-NSP5-BAP/D and F were obtained by PCR amplification of pCI-NSP5/D and F using specific primers to insert *Mlu*I/BAP tag and *Not*I sites, followed by ligation into those sites in pCI-Neo. pCI-NSP5 (1–178)-BAP/A, pCI-NSP5 (1–124)-BAP/B, pCI-NSP5 (1–150)-BAP/C, pCI-NSP5 (1–144)-BAP/G, pCI-NSP5 (1–151)-BAP/H, pCI-NSP5 (1–104)-BAP/I, and pCI-NSP5 (1–136)-BAP/J were obtained by PCR amplification of the respective pCI-NSP5-BAP using specific primers to insert indicated ORF deletions and *Mlu*I/*BspE*I restriction enzyme sites, followed by ligation into those sites in their respective in pCI-NSP5-BAP/A. pCI-BAP-NSP5 (15–195)/D and pCI-BAP-NSP5 (15–195)/F were obtained by PCR amplification of the respective pCI-BAP-NSP5 using specific primers to insert the BAP tag and *Mlu*I/*Not*I restriction sites, followed by ligation into those sites in pCI-Neo. pCI-NSP5-V5/A, B, C, G, H, I, and J pCI-NSP5∆T-V5/A, B, C, G, H, I, and J were obtained by annealing the following oligonucleotides 5'-ccggaggcaagcctattcctaaccctctgctgggcctggacagcacctaagc-3' and 5'-ggccgcttaggtgctgt ccaggcccagcagagggttaggaataggcttgcct-3' encoding a V5 tag and ligation between *BspE*I and *Not*I restriction sites of their respective pCI-NSP5-BAP or pCI-NSP5∆T. pCI-V5-NSP5/D and F were obtained by PCR amplification of their respective pCI-NSP5 using specific primers to insert *Mlu*I-V5 tag-*Not*I, followed by ligation between *Mlu*I and *Not*I in pCI-Neo. The chimeric NSP5∆T/H/TA, NSP5/H/TA, and NSP5∆T/J/TA were synthesized at GenScript as gene fragments and cloned in pCI-NSP5/A-BAP between *Mlu*I and *BspE*I restriction sites. The synthetic chimeric nucleotide sequences are available in the supplemental material.

pCI-NSP2-HA/A was obtained by PCR amplification of pcDNA-NSP2 (33) using specific primers to insert *Xho*I and HA tag/*Not*I, followed by ligation on those sites in pCI-Neo. pCI-NSP2-HA/B, C, D, F, G, H, I, and J were obtained by PCR amplification of their respective pCI-NSP2 using specific primers to insert *Mlu*I and HAtag/*Not*I, followed by ligation on those sites in pCI-Neo. The oligonucleotides were synthesized at Microsynth (Switzerland) and described in Table S3.

### AlphaFold predictions

Protein structures of NSP5 monomers, NSP5 dimers, or NSP2 multimers were predicted using AlphaFold3 Server (https://golgi.sandbox.google.com/about) (66).

### IDR prediction

The intrinsically disordered regions of proteins were determined using PONDR (Molecular Kinetics, Inc, http://www.pondr.com) using the VSL2 algorithm. Data were plotted with GraphPad Prism [version 10.1.1(270)].

### Immunofluorescence

MA/cytBirA cells were seeded at 1 × 10^5^ cells per well onto coverslips in a 24-well multiwell plate. The cells were infected with vvT7.3, followed by transfection using Lipofectamine 2000 (Thermo Fisher Scientific) as described previously (40). Specifically, cells were transfected with a ratio of NSP5 and NSP2 of 1:2 using 750 ng and 1,500 ng of DNA plasmids, respectively. The cells were supplemented with 100 µM biotin when the transfection mixture was added. LMH cells were seeded at a density of 3 × 10^5^ cells per well onto coverslips in a 24-well multiwell plate. The cells were transfected using *Trans*IT−2020 transfection reagent (Mirus Bio) according to the manufacturer’s instructions. Briefly, cells were transfected with a ratio of NSP5 and NSP2 of 1:2 using 750 ng and 1,500 ng DNA plasmids, respectively. For this purpose, 100 µL Opti-MEM (Thermo Fisher Scientific) was mixed with 3 µL of *Trans*IT−2020 and DNA plasmids and incubated for 15 min at room temperature. The cells were washed once with phosphate-buffered saline (PBS) and then added 400 µL of serum-free Waymouth’s MB725/1 medium per well, followed by the addition of 100 µL transfection mix. For nocodazole treatment, the medium was replaced 30 min before fixation with a medium containing 10 µM nocodazole (16). At 16 hpt, the medium was removed, and the cells were fixed with 2% paraformaldehyde for 10 min at room temperature or with ice-cold methanol for 3 min at −20°C. The cells were permeabilized with 0.1% Triton X-100-PBS for 5 min at room temperature and blocked in 1% bovine serum albumin (BSA)-PBS for 20 min at room temperature. The primary and secondary antibodies were diluted in 1% BSA-PBS and incubated for 40 min at room temperature in a humid chamber. The coverslips were mounted onto slides using ProLong Gold antifade mountant (Thermo Fisher Scientific).

Images were acquired using a confocal laser scanning microscope (DM550Q, Leica). Data were analyzed with Leica Application Suite (Mannheim; Germany) and ImageJ2 (version: 2.14.0/1.54f, http://imagej.net/Contributors).

### Co-immunoprecipitation and immunoblotting

1.2 × 10^6^ MA/cytBirA cells were infected with vvT7.3 at a multiplicity of infection (MOI) of 3 PFU/cell. Then, the cells were transfected with Lipofectamine 2000 (Thermo Fisher Scientific) in a ratio 1:1 of NSP5-BAP and NSP5-V5 or 1:2 of NSP5-BAP and NSP2-HA following the manufacturer’s instructions. After adding the transfection mixture, the cells were immediately supplemented with 100 µM biotin. At 16 hpt, the cells were lysed in 180 µL of TNN buffer [100 mM Tris-HCl, pH 8.0, 250 mM NaCl, 0.5% Nonidet P-40, and cOmplete protease inhibitor cocktail (Roche, Switzerland)] for 10 min on ice. For NSP5-BAP and NSP2-HA assays, the cells were crosslinked with 300 µM or 600 µM DSP prior to lysis as described in detail by Eichwald et al., 2004 (15). The cell lysates were clarified by centrifugation at 17,000 *× g* for 7 min and 4°C and then transferred to a new 1.5 mL tube. The input corresponded to 15 µL of cell lysate. For immunoprecipitation, the cell lysate was split into equal volumes and combined with 2 µg mouse mAb anti-V5 (SV5-PK1; Abcam, ab27671), mouse mAb anti-HA (clone HA-7; Merck, H3663), or 2 µg mouse IgG2a kappa isotype control (clone eBM2a; eBioscience, 14–4724-82) and incubated at 4°C for 30 min with rotation. The cell lysates were combined with 50 µL of Protein G Dynabeads (ThermoFisher, 10004D), equilibrated in TNN, and re-incubated at 4°C for 30 min with rotation. The bead-bound antibody-antigen complexes were washed four times with 500 µL TNN, eluted with SDS sample buffer, and resolved by SDS-PAGE. The proteins were detected by immunoblotting, as described below.

### Immunoblotting

Cells seeded in 12-well tissue culture plates at a density of 2 × 10^5^ cells per well were lysed directly by adding 25 µL of Laemmli sample buffer 4× (8% SDS, 40% glycerol, 200 mM Tris-HCl pH 6.8, and 0.4% bromophenol blue). The cell extracts were heated for 5 min at 95°C, sonicated for 5 s at 14 Hz, and loaded in SDS-PAGE. The proteins were separated by electrophoresis at 30 mA and then transferred to 0.45 µm Protan nitrocellulose membranes (Amersham). The membranes were blocked for 30 min in 5% milk-PBS and then incubated with primary and the corresponding secondary antibody conjugated to IRDye 680 or IRDye800 (LI-COR). For incubation with streptavidin-IRDye 800, the membrane was blocked and incubated with 1% BSA-PBS. Samples were acquired at Odyssey M Imager (LI-COR Biosciences).

### Phylogenetic tree analysis

The CDS for rotavirus NSP2 and NSP5 proteins were translated *in silico* into amino acids sequences using EMBOSS “transeq” (http://emboss.open-bio.org). The protein sequences were aligned using “mafft”' [MAFFT v7.475 (23 November 2020); https://mafft.cbrc.jp/alignment/software/] (81, 82), and the aligned protein sequences were back-translated using EMBOSS “tranalign” (http://emboss.open-bio.org). The phylogeny from the nucleotide multiple sequence alignments was then inferred by using “BEAUTi” and “BEAST” (v1.10.4) (83). In brief, 10,000,000 Markov chain Monte Carlo (MCMC) steps with the Juke-Cantor model were performed, saving each 10,000th tree. After the burn-in of 100,000 states, consensus trees for NSP2 and NSP5, respectively, were calculated and visualized using FigTree (https://beast.community).

## Data Availability

The GenBank accession numbers for NSP5 and NSP2 of RV species A to J used in this study are available in Table 1. The sequences of open reading frames of NSP5 and NSP2 of RV species A to J are described in the supplemental material.

## SUPPLEMENTAL MATERIAL

Fig.S1 jvi.00975-24-s0001.tiff Amino acid alignments of NSP5 and NSP2 of RV species A to J.

Fig. S2 jvi.00975-24-s0002.tiff AlphaFold3 predictions for NSP5 and NSP2.

Fig. S3 jvi.00975-24-s0003.tiff RVC-VLSs have filamentous morphology.

Fig. S4 jvi.00975-24-s0004.tiff Immunofluorescence images of MA/cytBirA cells co-expressing NSP5-BAP and NSP2-HA of species H and J at diverse ratios.

Fig. S5 jvi.00975-24-s0005.tiff NSP5-V5 in RV species A to J. Chimeric NSP5 H and J with NSP5 tail region of RVA.

Fig. S6 jvi.00975-24-s0006.tiff Heterologous formation of VLS between species A and other species.

Fig. S7 jvi.00975-24-s0007.tiff AlphaFold prediction for dimeric full-length NSP5 and NSP5∆T across RV species A to J.

Supplemental material jvi.00975-24-s0008.docx Figure legends; Tables S1 to S3; sequences.

ASM does not own the copyrights to Supplemental Material that may be linked to, or accessed through, an article. The authors have granted ASM a non-exclusive, world-wide license to publish the Supplemental Material files. Please contact the corresponding author directly for reuse.
