# Supplementary figures and images for: Characterization of viroplasm-like structures by co-expression of NSP5 and NSP2 across rotavirus species A to J
Source: J Virol. 2024 Aug 28;98(9):e00975-24. doi: 10.1128/jvi.00975-24 (PMC11423710; doi:10.1128/jvi.00975-24)

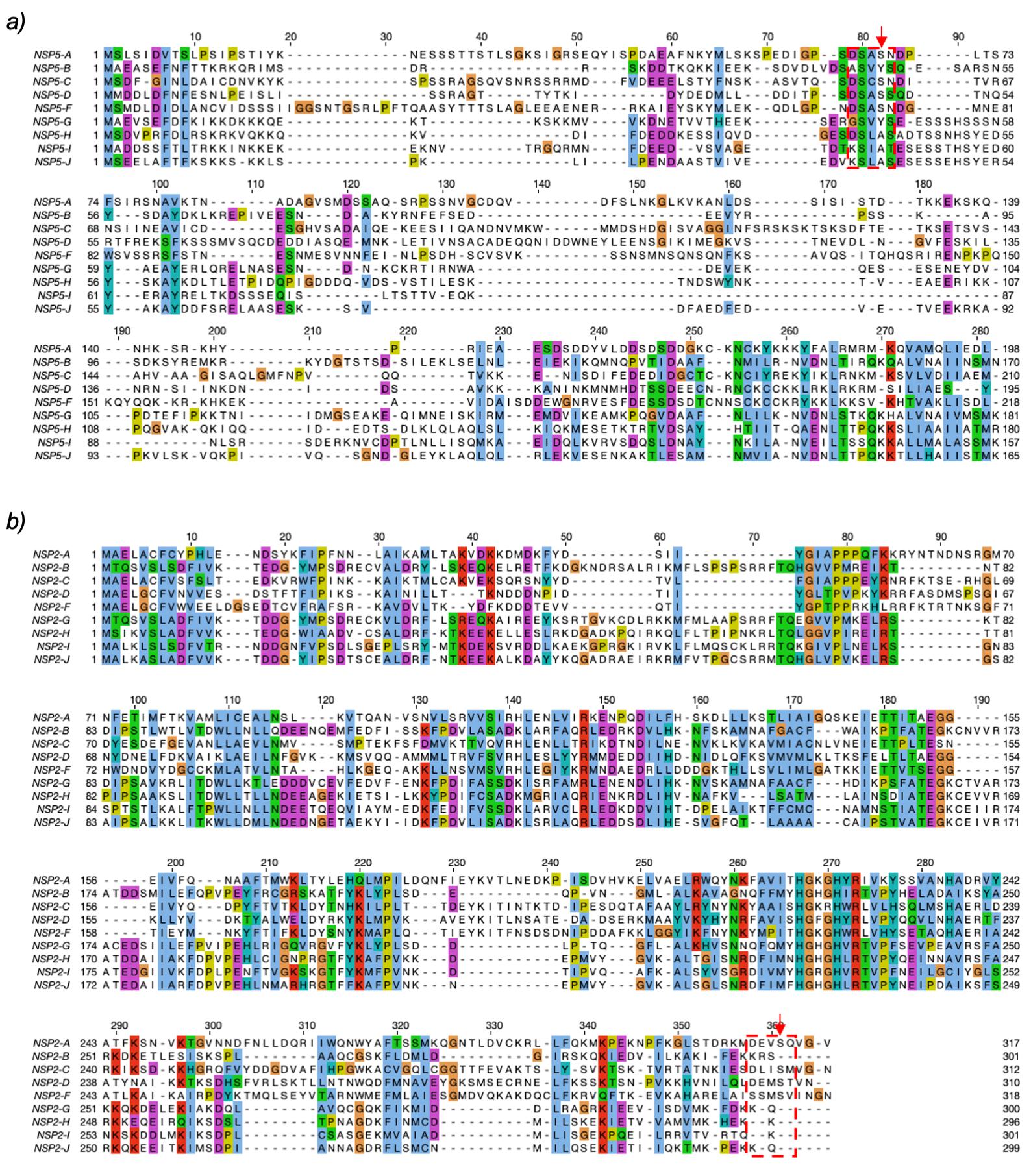

Supplement: Fig.S1 — Amino acid alignments of NSP5 and NSP2 of RV species A to J. [file jvi.00975-24-s0001.tiff]

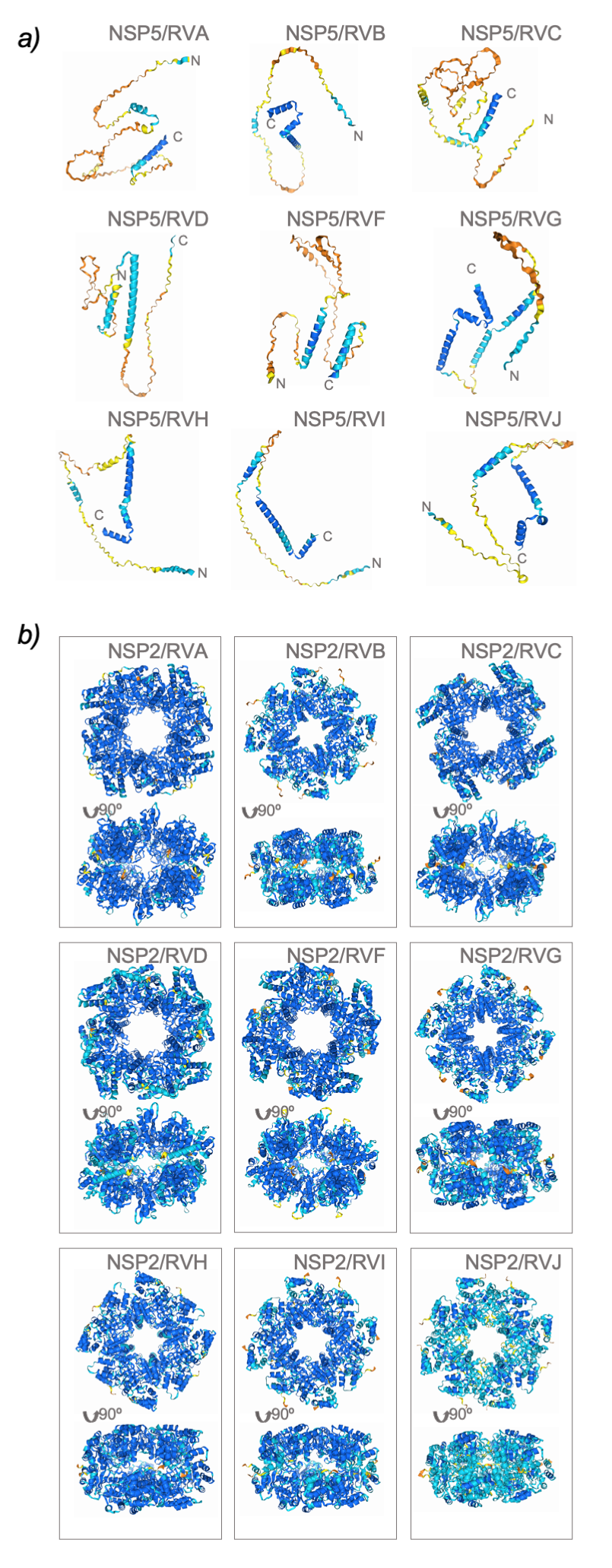

Supplement: Fig. S2 — AlphaFold3 predictions for NSP5 and NSP2. [file jvi.00975-24-s0002.tiff]

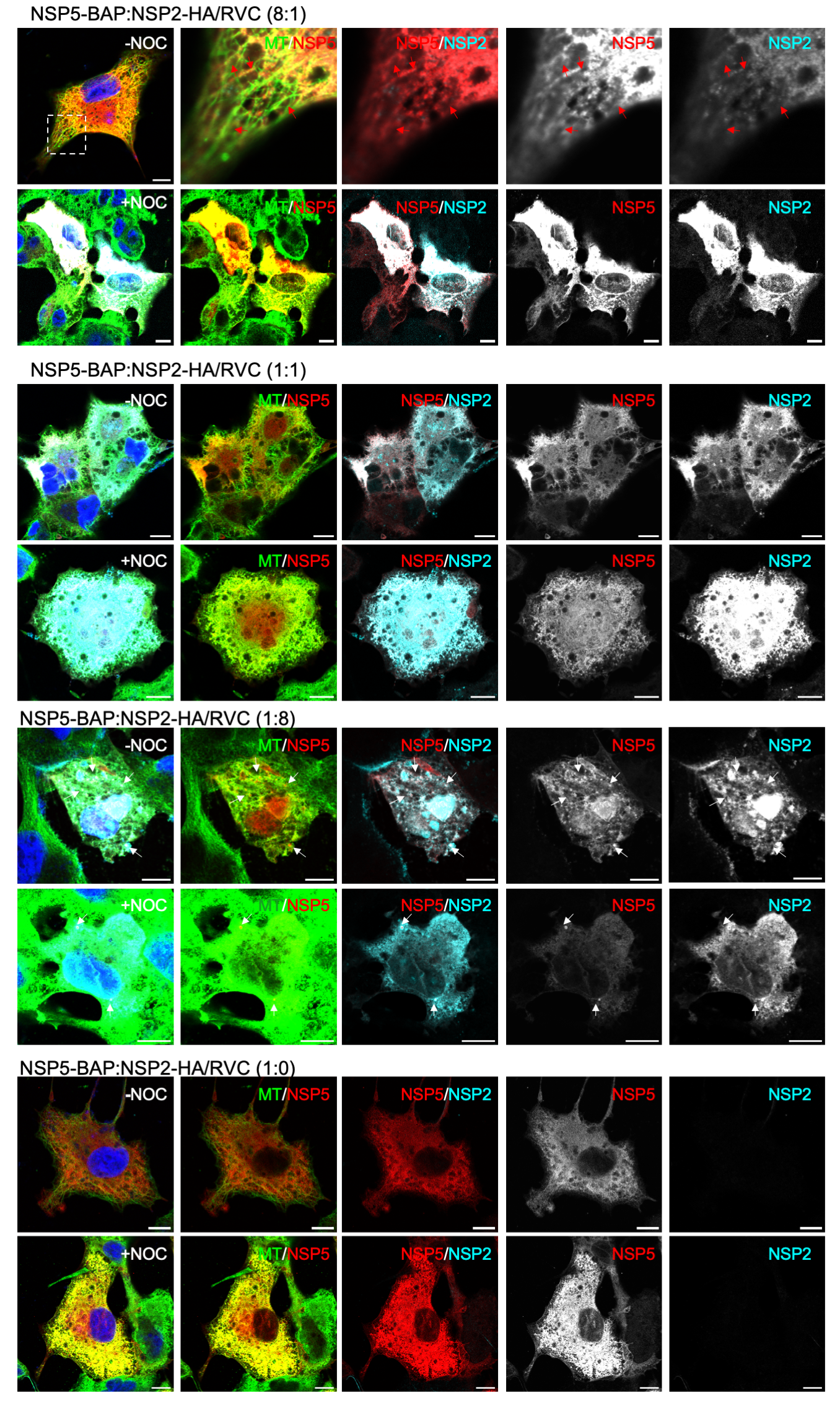

Supplement: Fig. S3 — RVC-VLSs have filamentous morphology. [file jvi.00975-24-s0003.tiff]

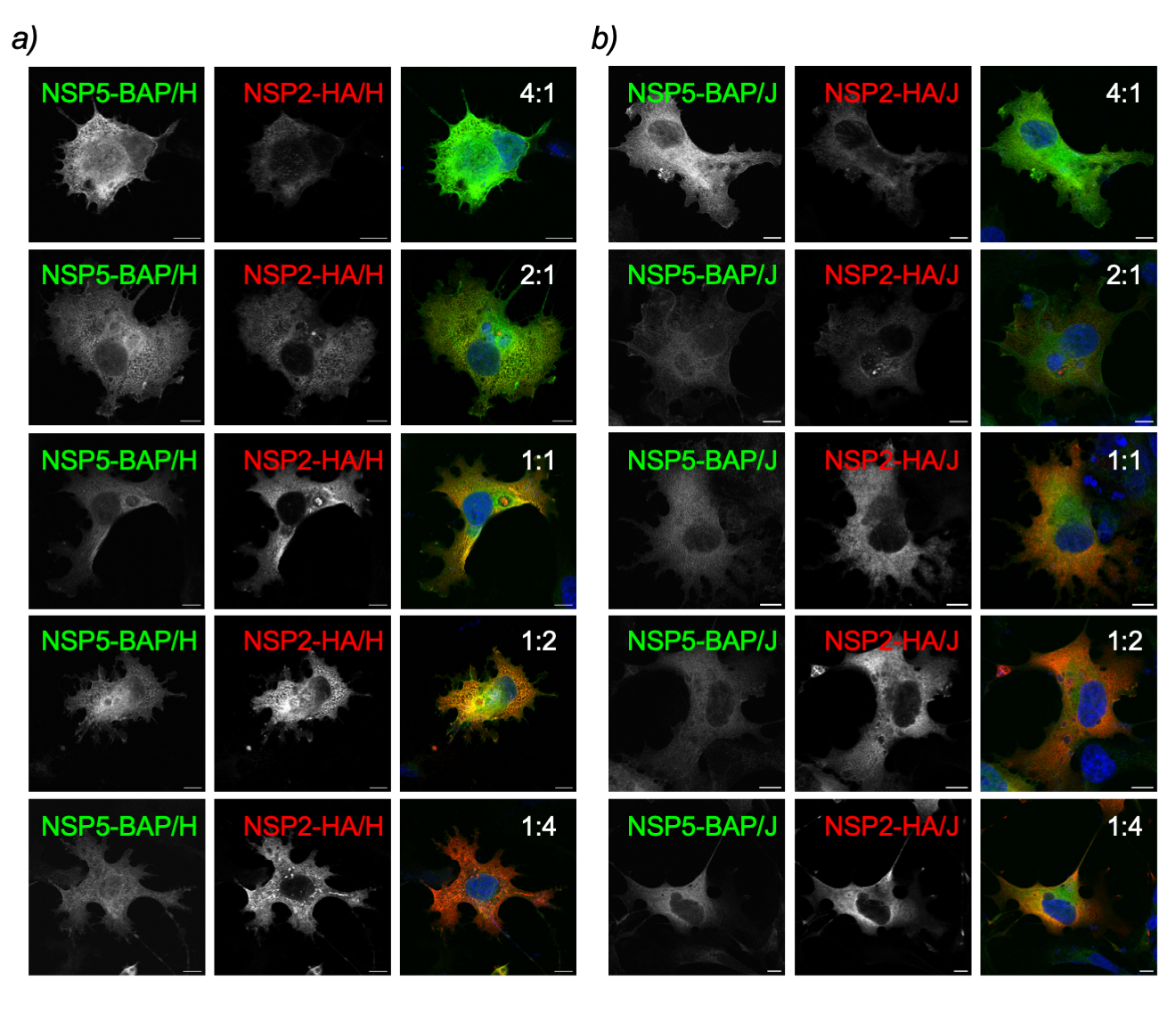

Supplement: Fig. S4 — Immunofluorescence images of MA/cytBirA cells co-expressing NSP5-BAP and NSP2-HA of species H and J at diverse ratios. [file jvi.00975-24-s0004.tiff]

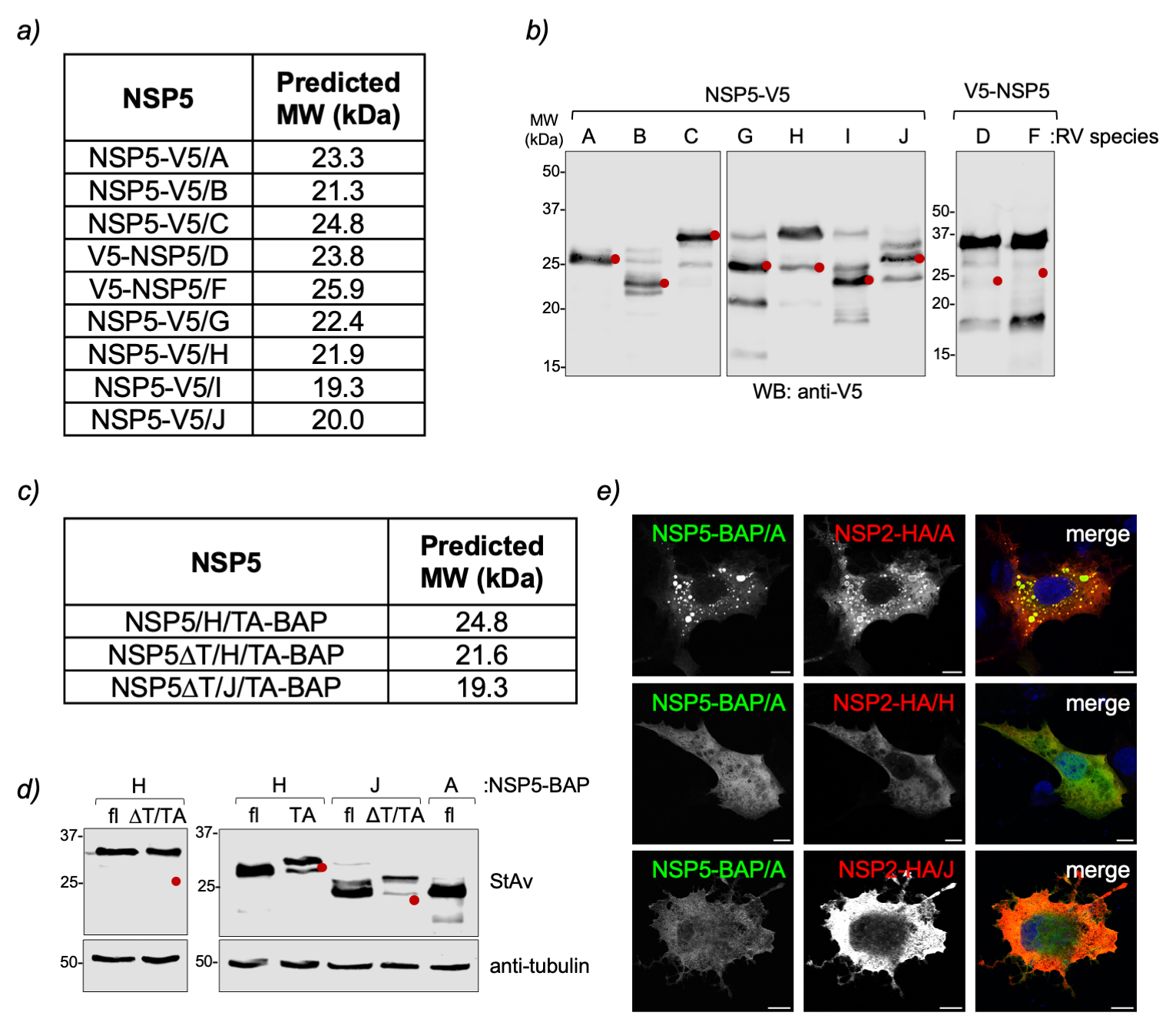

Supplement: Fig. S5 — NSP5-V5 in RV species A to J. Chimeric NSP5 H and J with NSP5 tail region of RVA. [file jvi.00975-24-s0005.tiff]

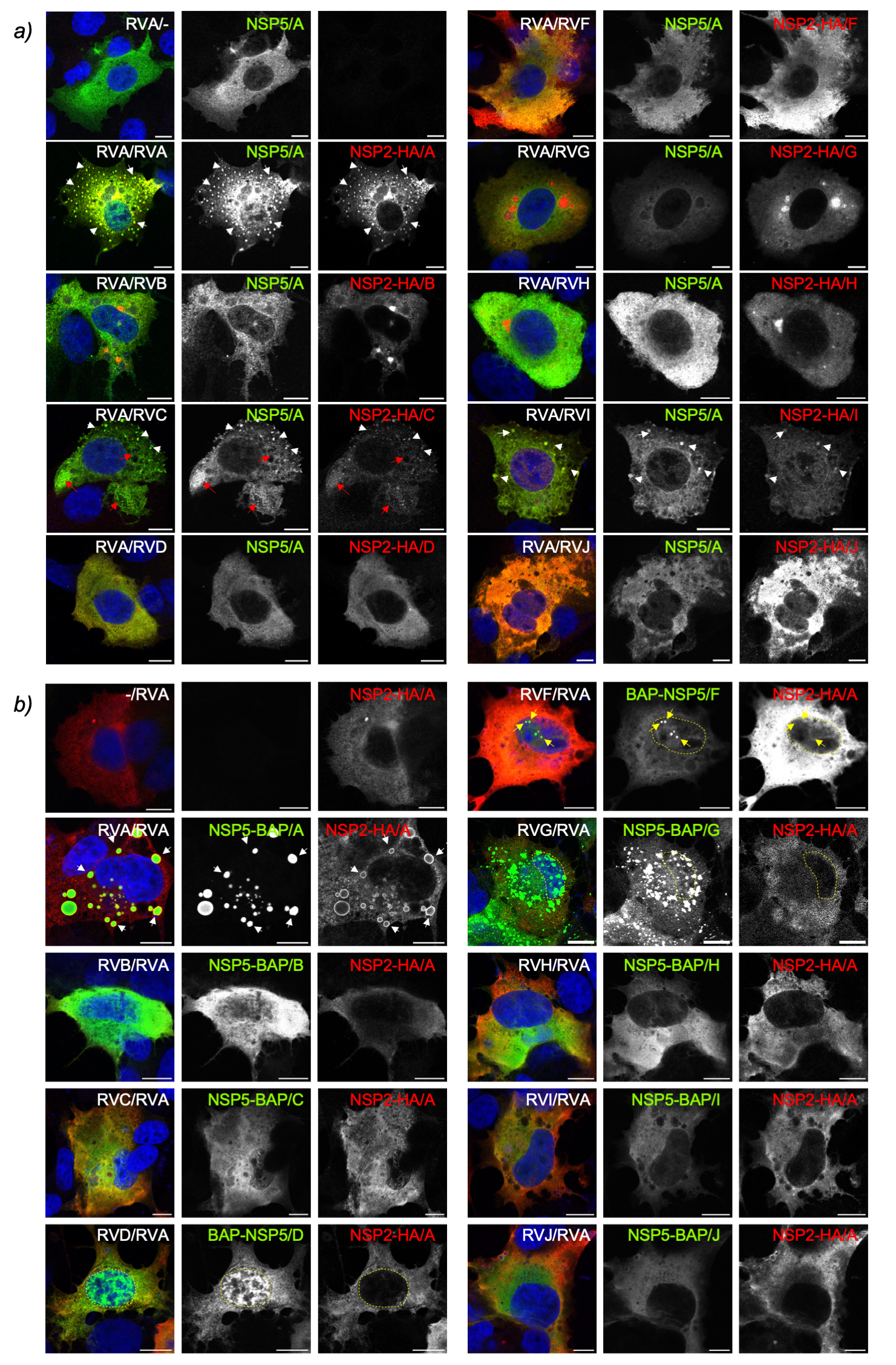

Supplement: Fig. S6 — Heterologous formation of VLS between species A and other species. [file jvi.00975-24-s0006.tiff]

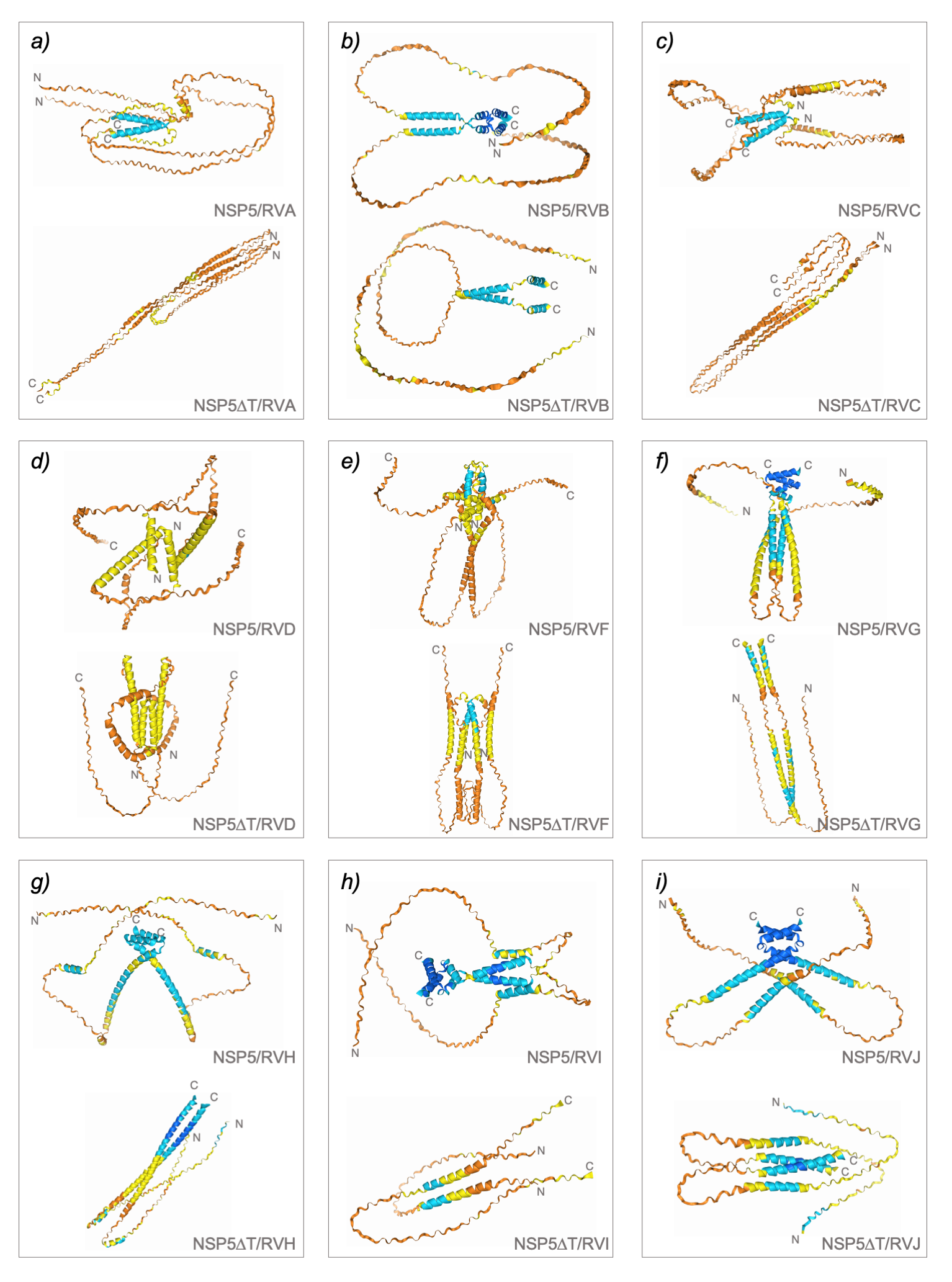

Supplement: Fig. S7 — AlphaFold prediction for dimeric full-length NSP5 and NSP5∆T across RV species A to J. [file jvi.00975-24-s0007.tiff]
