# Supplementary material for: Characterization of viroplasm-like structures by co-expression of NSP5 and NSP2 across rotavirus species A to J
Source: J Virol. 2024 Aug 28;98(9):e00975-24. doi: 10.1128/jvi.00975-24 (PMC11423710; doi:10.1128/jvi.00975-24)
Supplement: Supplemental material — Figure legends; Tables S1 to S3; sequences. [file jvi.00975-24-s0008.docx]

**SUPPLEMENTAL MATERIAL**

**Supplemental Figure Legends**

**Figure S1. Amino acid alignments of NSP5 and NSP2 of RV species A to J.** T-coffee alignment of NSP5 **(a)** and NSP2 **(b)** of RV species A to J. GenBank accession numbers are listed in Materials and Methods. The conserved residues are labeled according to the Clustal X classification, where blue is hydrophobic, red is positively charged, magenta is negatively charged, green is polar, pink is cysteine, orange is glycine, yellow is proline, cyan is aromatic, and white is unconserved. The first and last residues of each RV protein are indicated. For NSP5 **(a)** and NSP2 **(b)**, a discontinued red box highlights the non-canonical motif for casein kinase-1 alpha [(E,D)_n_-X-X-S/T-X], where the red arrows point to serine 67 and serine 313 of RVA NSP5 and NSP2, respectively.

**Figure S2****. AlphaFold3 predictions for NSP5 and NSP2. a)** Prediction of monomeric structure of NSP5. AlphaFold3 prediction of the monomeric structures of NSP5 in RV species A to J as indicated. **b)** Prediction of the octameric structure of NSP2. AlphaFold3 prediction of the octameric structures of NSP2 for RV species A to J. Upper (top) and side (bottom) views for each NSP2 RV species are presented in each panel. For top and bottom panels, the values of the predicted local distance difference test (pLDDT) are shown as color on each predicted structure: dark blue (pLDDT >90), light blue (pLDDT between 90 and 70), yellow (pLDDT between 70 and 50) and orange (pLDDT < 50).

**Figure S3. RVC-VLSs have filamentous morphology.** Immunofluorescence images of MA/cytBir cells co-expressing RVC NSP5-BAP and NSP2-HA at the indicated plasmid DNA ratios. The cells were untreated (-NOC) or treated (+NOC) with 10 µM nocodazole for 30 min before fixation. The cells were fixed with methanol at 16 hpt and immunostained for detection of NSP5-BAP (StAV, red), NSP2-HA (anti-HA, cyan), and microtubules (anti-tubulin-Alexa 488, green). Nuclei were stained with DAPI (blue). The scale bar is 10 µm. The dashed open square corresponds to the magnified images on the right for better visualization of filamentous VLSs. Red and white arrows point to filamentous and globular VLSs, respectively.

**Figure S4.** Immunofluorescence images of MA/cytBirA cells co-expressing NSP5-BAP and NSP2-HA of species H **(a)** and J **(b)** at the indicated plasmid DNA (NSP5:NSP2) ratios. At 16 hpt, the cells were fixed and immunostained for the detection of NSP5-BAP (StAV, green) and NSP2-HA (anti-HA, red). The nuclei were stained with DAPI (blue). The scale bar is 10 µm.

**Figure S5. a)** Table indicating the predicted molecular weights (kDa) of NSP5 fused to V5 tag of species A to J. **b)** Immunoblotting of extracts from MA104 cells expressing NSP5-V5 from species A to J, as indicated. The membrane was incubated with mAb anti-V5. The red point indicates the predicted MW of each NSP5-V5 across indicated RV species. **c)** Table indicating the predicted molecular weights (kDa) of chimeric NSP5 H and J with BAP tag. **d)** Immunoblotting comparing extracts from MA/cytBirA cells expressing chimeric NSP5 H and J having fused the tail region of NSP5/A as indicated. The NSP5 chimeric proteins are fused at their C-terminal to a BAP tag. The cell extracts were prepared at 16 hpt. The membrane was incubated with streptavidin-IRDye800. Anti-tubulin was used as a loading control. **e)** Immunofluorescence images of MA/cytBirA cells co-expressing NSP5-BAP/A with NSP2-HA A, H, and J. At 16 hpt, the cells were fixed and immunostained for the detection of NSP5-BAP (StAv, green) and NSP2-HA (anti-HA, red). Nuclei were stained with DAPI (blue). The scale bar is 10 µm.

**Figure S6. Heterologous formation of VLS between species A and other species.** **a)** Immunofluorescence images of MA104 cells expressing NSP5 from RV species A without or with NSP2-HA of species A to J. At 16 hpt, the cells were fixed and immunostained for detection of NSP5/A (guinea pig anti-NSP5, green, middle column) and NSP2-HA/A to J (anti-HA, red, right column). Nuclei were stained with DAPI (blue). A merged image is presented in the left column. The letters in the upper right corner of the merged image correspond to the RV species of NSP5 and NSP2, respectively. The scale bar is 10 µm. **b)** Immunofluorescence images of MA/cytBirA cells expressing NSP2-HA/A without and with NSP5-BAP/A to J. The cells were fixed and immunostained to detect NSP5-BAP (StAV, red, middle column) and NSP2-HA (anti-HA, red, left column). Nuclei were stained with DAPI (blue). A merged image is presented in the left column. The letters in the upper right corner of the merged image correspond to the RV species of NSP5 and NSP2, respectively. The scale bar is 10 µm. The white arrows point to VLSs, the yellow arrows point to nuclear inclusions, and the yellow line labels the nucleus as determined by the DAPI signal.

**Figure S7. AlphaFold prediction for dimeric full-length NSP5 and NSP5∆T across RV species A to J.** Full-length NSP5 and NSP5∆T structural dimeric association was predicted employing AlphaFold3. Each panel represents a predicted full-length (top) and ∆T deletion mutant (bottom) of NSP5 dimers of species A (**a**), B(**b**), C(**c**), D(**d**), F(**e**), G(**f**), H(**g**), I(**h**), and J(**i**). The values of the predicted local distance difference test (pLDDT) are shown as color on each predicted structure: dark blue (pLDDT >90), light blue (pLDDT between 90 and 70), yellow (pLDDT between 70 and 50) and orange (pLDDT < 50).

**Open-reading frame sequences of NSP5 and NSP2 of species B to J used in this study.**

Start and stop codons are highlighted in bold. The Kozak sequence, added at the 5’ end of each ORF, used in this study is labeled in italics

**Rotavirus B**

**NSP5-RVB**

>AFE206724.1 Human rotavirus B strain CAL-1

*GCCACC****ATG***GCAGAAGCGTCTGAGTTCAACTTTACCACAAAAAGAAAGCAGAGAATAATGAGTGATCGAAGATCAAAAGATGACACAAAACAAAAAAAGATTGAAGAAAAATCTGATGTTGACTTGGTTGATTCAGCCTCAGTTTATTCACAAGAATCTGCCAGAAGTAACTATAGTGATGCTTACGATAAGTTAAAACGTGAACCAATAGTTGAAGAATCAAATGACGCGAAGTATAGGAATTTTGAATTTTCTGAAGATGAAGAAGTTTATAGACCATCAAGTAAAGCGTCAGACAAATCATATAGAGAAATGAAACGTAAATATGATGGTACTAGCACGTCAGATTCCATTTTAGAAAAATTGTCAGAGTTAAATCTCGAGATTGAGAAGATAAAACAAATGAACCAACCAGTCACAATCGATGCAGCATTCAACATGATTCTTCGAAACGTTGACAATCTAACTATAAGGCAGAAACAGGCATTAGTAAACGCAATTATTAATTCTATGAAC**TAG**

**NSP2-RVB**

>AF205850.1 Human rotavirus B strain CAL-1

*GCCACC****ATG***ACGCAATCAGTTTCTCTTTCTGATTTCATCGTTAAGACTGAAGATGGATATATGCCATCAGACAGAGAATGTGTTGCATTGGATAGATATTTATCCAAAGAGCAGAAGGAACTAAGAGAAACTTTTAAGGATGGAAAAAATGATAGATCAGCTTTAAGAATTAAAATGTTCTTATCTCCTTCACCTTCCAGACGATTCACTCAACATGGAGTTGTTCCAATGAGAGAAATAAAAACAAATACGGATATACCAAGTACACTATGGACTCTTGTGACTGATTGGTTACTAAATTTACTTCAAGATGAAGAAAATCAGGAAATGTTTGAAGATTTTATTAGTTCCAAATTTCCGGATGTTTTAGCTTCGGCAGACAAGCTAGCCCGTTTCGCCCAGCGATTGGAGGATAGAAAAGATGTGTTGCACAAGAATTTTTCTAAAGCCATGAATGCCTTTGGCGCCTGTTTTTGGGCGATCAAACCGACTTTTGCCACTGAGGGAAAATGTAATGTTGTAAGAGCCACTGATGATTCAATGATACTAGAGTTTCAGCCAGTACCAGAATATTTTCGTTGTGGAAGATCAAAAGCTACATTTTATAAATTATACCCACTCTCAGACGAACAACCCGTCAATGGCATGCTCGCTCTAAAAGCAGTTGCTGGAAATCAGTTTTTTATGTACCATGGACATGGGCACATCAGAACAGTTCCATATCATGAACTTGCTGATGCCATCAAATCATACGCTCGTAAAGATAAAGAAACGCTCGAGAGCATTTCTAAATCACCACTTGCAGCTCAATGTGGTAGTAAATTTCTCGATATGCTCGATGGAATTAGATCAAAACAAAAAATTGAAGATGTGATTTTGAAAGCAAAAATTTTTGAAAAGAAAAGAAGC**TGA**

**Rotavirus C**

**NSP5-RVC**

>KP982878.1 Rotavirus C NSP5

*GCCACC****ATG***TCTGATTTCGGAATTAATCTTGATGCCATTTGCGACAATGTTAAATATAAATCACCAAGTTCAAGAGCAGGATCTCAAGTATCAAATCGGAGTTCAAGAAGGATGGATTTTGTAGATGAAGAAGAGCTGAGCACTTATTTTAATTCAAAAGCATCAGTGACACAATCAGATTCATGTTCCAATGATATAACGGTTAGGAATTCAATTATCAATGAGGCTGTAATATGCGATGAATCAGGACATGTGTCAGCTGATGCAATCCAGGAGAAAGAAGAATCCATTATACAAGCAAATGACAATGTGATGAAGTGGATGATGGATTCACATGATGGTATTAGTGTGGCTGGGGGAATAAATTTTTCAAGATCAAAGAGCAAGACAAGTAAAAGTGATTTTACAGAAACCAAATCAGAAACAAGTGTCTCAGCTCATGTTGCGGCAGGAATTAGCGCACAGTTAGGAATGTTCAATCCAGTTCAACAAACTGTTAAGAAGGAGAATATATCTGATATTTTTGAAGATGAAGATATTGATGGATGTACTTGCAAAAATTGCATATATAGAGAAAAATACATCAAACTCCGAAATAAAATGAAAAGTGTTTTGGTTGATATTATTGCTGAAATG**TAA**

**NSP2-RVC**

>KP982875.1 RVC/Pig-wt/BEL/12R021/2012/G3P5_NSP2

*GCCACC****ATG***GCCGAGCTAGCCTGTTTCGTTTCCTTCTCACTGACTGAAGATAAGGTAAGATGGTTCCCGATCAACAAAAAAGCTATCAAAACAATGTTGTGTGCAAAAGTGGAGAAGAGTCAAAGAAGCAACTATTATGATACAGTCCTATTTGGTATTGCACCACCCCCTGAATATAGAAACAGATTTAAGACGAGTGAAAGGCATGGATTGGATTATGAATCTGATGAATTTGGTGAAGTTGCTAATTTGCTAGCAGAAGTCTTAAATATGGTATCAATGCCAACAGAAAAGTTCTCATTTGATATGGTCAAAACAACAGTTCAAGTTAGACATCTCGAGAATTTATTGACCAGAATTAAGGATACTAATGACATATTAAATGAGAATGTGAAATTAAAAGTAAAAGCAGTTATGATAGCATGCAACCTAGTTAATGAAATAGAAACTACTCCGTTAACTGAAAGCAATGAAATAGTGTATCAAGATCCGTATTTTACAGTAACCAAGTTAGATTACACCAATCATAAGATATTACCTTTAACAACAGATGAGTATAAAATTACAATTAATACTAAAACTGACATTCCAGAAAGTGATCAAACAGCTTTTGCAGCATATCTTAGATATAATTATAATAAATATGCAGCAATTTCACATGGGAAACGACATTGGAGATTAGTTCTTCATTCACAATTGATGTCACATGCTGAAAGGTTAGACAGAAAAATCAAATCAGATAAAAAACATGGTAGACAGTTTGTGTATGACGATGGAGATGTCGCCTTCATACATCCAGGATGGAAAGCATGTGTAGGTCAACTGTGTGGTGGCACAACATTTGAAGTAGCAAAAACATCATTATACAGTGTAAAGACATCAAAAACAGTGCGCACAGCAACAAACAAAATTGAAAGTGATTTAATTTCAATGGTTGGAAAT**TAG**

**Rotavirus D**

**NSP5-RVD**

>NC_014521.1 Rotavirus D chicken/05V0049/DEU/2005

*GCCACC****ATG***ATGGATGATTTAGACTTTAATTTTGAATCTAATCTACCTGAAATATCTTTAATTTCGTCACGAGCTGGAACAACATATACGAAAATAGATTATGATGAAGATATGTTGTTAGATGATATTACTCCATCAGATTCAGCATCATCACAGGATACTAATCAGAGAACTTTTAGAGAAAAATCTTTTAAATCTTCATCTATGGTTTCACAGTGTGATGAAGACGATATTGCTAGTCAAGAAATGAACAAATTAGAAACCATCGTTAATAGCGCATGTGCAGATGAACAACAAAACATTGATGATTGGAACGAATATCTAGAGGAAAATAGCGGTATTAAGATTATGGAAGGAAAAGTTTCTACAAATGAAGTTGATTTGAATGGTGTGTTTGAGTCTAAGATATTAAACCGCAACTCAATAATTAATAAAGATAATATTGATAGCGCTGTCAAGAAGAAGGCAAATATTAATAAGATGAATATGCATGATACGTCGTCTGATGAAGAATGCAATCGTAACTGCAAATGCTGTAAAAAATTGAGAAAATTAAGAAAACGTATGAGTATTTTAATTGCTGAAAGTTAT**TAA**

**NSP2-RVD**

>NC_014518.1 Rotavirus D chicken/05V0049/DEU/2005

*GCCACC****ATG***GCGGAGCTGGGCTGCTTCGTAAATGTGGTTGAATCTGATTCGACGTTTACATTCATTCCAATAAAATCAAAAGCTATTAATATATTGCTCACAACGAAAAATGATGATGACAATCCAATTGATACTATTATTTATGGTCTAACGCCGGTCCCAAAGTATAAAAGAAGATTTGCATCTGACATGAGTCCATCAGGTATTAATTATGATAATGAATTATTCGATAAAGTTGCTATTAAATTAGCGGAGATTCTGAATTTTGGGGTTAAAAAAATGTCAGTACAGCAAGCAATGATGATGCTAACAAGAGTGTTTTCAGTTAGACATCTGGAATCATTGTACTATAGAATGATGGACGAAGATGATATAATTCATGATAATATTGATCTACAATTCAAATCAGTTATGGTGATGCTGAAGTTAACCAAATCGTTCGAATTGACTCTAACTGCTGAAGGGGGGAAATTACTTTATGTTGATAAAACTTATGCACTTTGGGAACTAGACTATAGAAAGTATAAACTGATGCCTGTTAAAGCTGTTGAATATAAAATAACTTTAAATTCCGCAACAGAAGATGCGGATAGTGAAAGGAAAATGGCTGCATATGTTAAATACCATTATAATAGATTTGCTGTAATATCACATGGATTTGGTCATTATAGACTAGTTCCCTATCAGCAGGTACTAAATCATGCTGAACGTACTTTTGCGACATATAATGCAATCAAAAAGACGAAATCTGATCACTCATTTGTGCGACTTTCTAAAACTTTGTTGAATACAAATTGGCAAGATTTTATGAATGCTGTTGAATATGGAAAAAGCATGAGTGAATGTAGGAATGAATTATTTAAGTCATCAAAAACGAGTAATCCAGTCAAGAAGCACGTCAATATACTGCAACTGGATGAAATGTCCACTGTGAAT**TAG**

**Rotavirus F**

**NSP5-RVF**

>NC_021629.1 Rotavirus F chicken/03V0568/DEU/2003

*GCCACC****ATG***AGCATGGATCTTGATATAGACTTAGCCAACTGTGTTATTGATTCAAGTTCAATAATAGGAGGATCTAATACTGGATCTAGATTACCTTTTACGCAAGCAGCATCTTACACTACCACATCTCTTGCTGGTTTGGAGGAAGCGGAGAATGAACGAAGAAAGGCAATAGAGTATTCAAAATATATGCTAGAAAAACAAGATTTAGGTCCAAATGATTCCGCTTCGAACGACGGAATGAATGAATGGTCAGTATCATCAAGATCTTTTTCTACAAATGAGTCAAATATGGAATCTGTTAATAATTTTGAAATTAACCTACCGTCTGACCATTCTTGTGTTTCAGTTAAGTCATCTAATTCGATGAATTCACAAAATTCACAAAATTTCAAGTCCGCAGTGCAAAGCATTACTCAGCATCAATCACGAATTAGGGAGAATCCTAAACCACAAAAGCAGTATCAACAAAAGAAGAGGAAACATAAAGAAAAGGCTGTGATTGATGCCATTTCTGATGATGAATGGGGAAATAGAGTAGAGTCATTTGATGAATCATCGGATTCAGACACCTGTAATAATAGTTGTAAGTGTTGCAAGCGCTACAAGAAACTAAAGAAAAGTGTGAAGCATACAGTGGCTAAGCTTATATCCGATCTA**TAA**

**NSP2-RVF**

>JQ920000.1 Rotavirus F chicken/03V0568/DEU/2003

*GCCACC****ATG***GCCGAGCTGGGTTGTTTCGTTTGGGTTGAGGAATTGGATGGCTCAGAAGATACTTGTGTCTTTAGGGCGTTTAGTCGTAAAGCAGTTGATGTGCTTACAAAATACGATTTTAAGGATGATGATACTGAAGTTGTGCAAACAATTTATGGACCAACACCACCTAGAAAGCATCTTCGTAGGTTTAAGACTAGAACGAACAAATCAGGATTTCACTGGGATAATGATGTTTATGACGGATGCTGTAAGATGTTGGCTACTGTTTTAAATACTGCACATCTTAAAGGAGAACAAGCTAAGAAATTGTTGAATAGTGTTATGTCAGTAAGGCATCTTGAGGGAATATATAAAAGAATGAATGATGCAGAAGATAGGCTACTGGATGATGATGGTAAGACTCATCTCTTGTCTGTACTAATTATGCTTGGGGCAACGAAGAAAATTGAGACTACTGTAACATCAGAAGGTGGTACTATTGAATATATGAATAAGTACTTCACGATATTCAAATTAGACTATTCAAATTATAAAATGGCGCCACTGCAAACAATTGAGTATAAAATTACATTCAATAGTGATTCAGACAACATACCAGATGATGCGTTTAAAAAACTAGGTGGATATATTAAATTTAATTACAACAAGTACATGCCAATAACACATGGTAAAGGACACTGGAGACTAGTTCACTACTCAGAGACTGCGCAACACGCTGAGAGAATAGCTGCGACTCTTAAAGCTATTAAAGCAATAAGACCAGACTATAAAACAATGCAGCTTTCTGAATATGTTACTGCTAGGAATTGGATGGAATTTATGCTAGCAATAGAGTCTGGTATGGATGTACAAAAAGCAAAAGATCAATGCTTGTTTAAGCGTGTTCAATTTACTAAAGAAGTCAAAGCACATGCTAGAGAACTCGCTATATCATCTATGTCTGTTATAAACGGAAAT**TGA**

**Rotavirus G**

**NSP5-RVG**

>JQ920012.1 Rotavirus G chicken/03V0567/DEU/2003

*GCCACC****ATG***GCTGAAGTCTCTGAATTTGATTTCAAAATAAAGAAAGATAAGAAGAAACAAGAAAAAACGAAATCGAAGAAAATGGTTGTCAAAGATAATGAAACAGTAGTAACACATGAAGAAAAGTCTGAACGTGGTTCAGTCTATTCTGAAGAATCATCAAGTCATTCATCAAGTAATTACGCTGAAGCATATGAAAGGCTACAGCGTGAACTTAATGCTAGCGAATCAAATGACAACAAGTGTAAGCGAACAATTAGAAACTGGGCAGATGAAGTTGAAAAACAAGAAAGTGAATCTGAGAACGAATATGATGTACCAGATACAGAATTCATTCCAAAGAAAACAAACATAATAGATATGGGGTCTGAAGCAAAAGAGCAGATAATGAATGAAATATCAAAAATTAGAATGGAAATGGATGTAATTAAAGAAGCAATGAAACCACAAGGCGTTGATGCTGCATTTAACCTCATTCTCAAGAATGTAGACAACCTATCTACAAAACAAAAACACGCTCTTGTAAACGCAATAGTTATGTCAATGAAA**TAA**

**NSP2-RVG**

>JQ920009.1 Rotavirus G chicken/03V0567/DEU/2003

*GCCACC****ATG***ACGCAGTCGGTTTCATTAGCTGATTTCATTGTCAAAACTGATGATGGTTATATGCCAAGTGACAGAGAATGTAAAGTGCTTGATAGATTTCTGTCCAGAGAACAAAAAGCAATTCGTGAAGAATATAAATCCAGAACGGGAGTGAAATGTGATTTAAGAAAGAAAATGTTTATGCTAGCTGCACCATCAAGGAGATTTACTCAGGAAGGTGTAGTTCCTATGAAGGAATTGAGAAGTAAGACTGACATTCCAAGTGCAGTGAAAAGATTGATCACTGATTGGCTGCTGAAAACACTAGAAGACGACGATGTCTGTGAAGTTTTTGAAGATGTATTTGAAAATAAGTTTCCAGATATTTTCGCATCATCGGATAAAATTTCAAGATTTGCAATGCGACTAGAAAATGAAAACGATTTGATTCATAAAAATGTATCAAAGGCAATGAATGCGTTTGCAGCTTGTTTTCACGATATTAAGCCTTCATTTGCCACAGAGGGAAAATGTACTGTTGCAAGAGCATGTGAAGATTCGATCATTCTGGAATTTCCTGTAATACCAGAACATCTGAGAATTGGACAAGTTAGAGGAGTATTCTATAAATTGTATCCACTATCTGATGATCTACCAACGCAAGGTTTTCTTGCTTTAAAACATGTGTCAAACAATCAATTCCAAATGTACCATGGACACGGACATGTTAGAACAGTACCGTTTTCTGAAGTGCCAGAAGCAGTACGATCATTCGCAAAAAAACAGAAAGATGAACTGGAAAAGATAGCTAAAGATCAACTTGCCGTGCAGTGCGGACAGAAATTCATTAAAATGATTGATGATCTACGTGCTGGTAGGAAGATTGAAGAAGTGATTTCTGACGTGATGAAATTCGACAAGAAACAG**TGA**

**Rotavirus H**

**NSP5-RVH**

>MT644988.1 Rotavirus H isolate SP-VC36

*GCCACC****ATG***AGCGACGTGCCTCGATTTGATCTCAGAAGTAAAAGGAAAGTACAAAAGAAGCAGAAAGTTGATATTTTTGACGAAGACGATAAGGAATCATCAATTCAGGTTGACGGTGAATCAGATTCATTAGCTTCAGCGGATACAAGTTCAAATCATAGTTACGAAGATTATTCAAAAGCTTATAAAGATCTAACCTTAGAGACACCAATTGACCAGCCAATTGGAGATGACGATCAAGTTGACTCTGTTTCGACCATTCTTGAAAGTAAGACTAATGATTCGTGGTATAATAAAACTGTTGAAGCTGAAGAACGAATTAAAAAACCGCAAGGGGTAGCTAAACAAAAGATCCAACAAATTGATGAAGATACATCTGATTTAAAGCTTCAACTAGCACAGCTCTCACTAAAAATACAAAAAATGGAATCAGAAACAAAAACCAGAACAGTCGATTCAGCATATCACACAATTATAACTCAGGCGGAAAATTTAACCACTCCACAGAAAAAATCGTTGATAGCAGCAATAATTGCAACAATGAGA**TAA**

**NSP2-RVH**

>MT644956.1 Rotavirus H isolate SP-VC36

*GCCACC****ATG***TCGATTAAAGTGTCACTTGCTGATTTCGTTGTAAAAACTGAAGATGGATGGATTGCTGCTGACGTTTGCTCAGCACTTGACAGATTTAAGACTAAAGAAGAGAAAGAGCTTTTGGAATCATTAAGAAAGGATGGAGCAGATAAGCCTCAGATCAGAAAACAGCTTTTCTTAACTCCAATTCCGAACAAGAGGTTAACACAGTTGGGTGGTGTACCAATCAGAGAAATTAGAACCACAACTCCAATTCCATCAGCCGCGAAATCACTTATTACTGATTGGTTGTTAACTCTACTAAATGATGAAGAGGCTGGCGAGAAGATAGAAACAAGTATTCTTAAGAAATATCCTGATATTTTTTGCTCAGCAGACAAAATGGGTAGAATCGCTCAGAGAATTGAGAACAAGCGTGATCTAATTCATGTTAATGCGTTTAAAGTGTTATCTGCAACGATGCTTGCAATTAATTCTGATATTGCAACTGAAGGAAAGTGTGAAGTAGTCAGAGCGACTGATGATGCAATTATTGCTAAATTCGATCCAGTTCCAGAACATCTATGTATTGGGAATCCAAGAGGTACATTCTATAAAGCGTTTCCCGTTAAAAAAGATGAACCGATGGTTTATGGTGTTAAAGCACTGACTGGAATTTCAAATAGAGATTTTATCATGAATCATGGACATGGACATTTAAGAACTGTACCATATCAGGAAATAAATAATGCAGTTAGATCATTTGCGAGGAAAAAAGAGCAGGAAATTAGACAAATTAAATCTGACTCACTAACACCAAATGCTGGAGATAAATTTATTAACATGTGTGATATGATTCTGTCAAAAGAGAAAATTGAAACTGTAGTTGCTATGGTTATGAAACATGAGAAGAAA**TAG**

**ROTAVIRUS I**

**NSP5-RVI**

>OM451078.1 MAG: Rotavirus I isolate SD-MO2

*GCCACC****ATG***GCAGACGATTCCAGCTTCACCTTGACAAGAAAGAAAATTAATAAAAAAGAGAAAGAAAAGAACGTCACAAGAGGTCAAAGAATGAATTTTGACGAAGAAGATGTTTCAGTAGCCGGTGAAACTGATACTAAGAGCATTGCTACAGAATCAGAGTCATCTACGCATTCATACGAAGACTACGAGAGAGCGTATAGAGAATTAACAAAAGATTCTAGTTCAGAACAAATAAGCTTAACTAGTACCACTGTTGAACAAAAGAATCTTTCTCGATCTGATGAGCGAAAAAATGTTTGTGATCCAACATTGAATTTACTAATCTCACAGATGAAAGCTGAAATTGATCAATTGAAAGTCAGAGTAAGTGATCAAAGTTTAGATAATGCTTATAATAAAATCCTTGCAAACGTAGAAATCTTGACCTCATCACAAAAAAAGGCTCTGCTAATGGCGCTAGCATCCTCAATGAAA**TAA**

**NSP2-RVI**

>OM451075.1 MAG: Rotavirus I isolate SD-MO2

*GCCACC****ATG***GCGCTGAAACTGAGTTTATCCGACTTTGTGACAAGAAATGATGATGGTAATTTTGTTCCAAGTGATTTGAGTGGAGAACCTTTGAGTAGATATATGACTAAAGATGAAAAATCAGTTAGAGATGATTTGAAAGCAGAAAAAGGACCAAGAGGAAAGATCAGAGTTAAACTCTTTCTAATGCAGTCTTGTAAATTGAGAAGGACACAGAAAGGCATTGTTCCTCTTAACGAATTGAAATCTGGAAATAGCCCAACATCTACTTTGAAAGCTCTTTTTACACCGTGGTTGCTTAATCTACTTAATGACGAAGAAACTCAAGAACAAGTGATTGCTTACATGGAAGATAAATTTGAAGATATTTTTGTTTCTTCTGATAAATTGGCTAGAGTTTGTCTTAGACTTGAAGATAAGGATGACGTTATTCACACAGATCCAGAATTGGCGATTAAAACATTTTTTTGTATGTGTAATGCAATGAATTCAACAATCGCAACAGAAGGAAAATGTGAAATTATAAGAGCAACTGAAGATGGAATAATTGTCAAATTTGACCCTCTACCTGAAAATTTCACAGTTGGTAAATCGAAAGGTACTTTTTACAAAATGTTTCCAGTTAATAAAGACACTATCCCAGTTCAAGCATTTAAAGCACTATCTTATGTTAGTGGGAGGGACATTGTCATGTATCATGGACGAGGACACGTGAGAACAGTTCCTTTCAATGAAATCCTTGGATGTATTTATGGACTATCAAATAAATCGAAAGATGATTTGATGAAAATTAAGTCTGATCCTCTTTGCTCGGCTTCTGGTGAAAAAATGGTTGCAATAGCTGATATGTTGATTTCAGGTGAAAAACCACAAGAAATACTTCGACGAGTGACTGTAAGAACACAGAAA**TGA**

**ROTAVIRUS J**

**NSP5-RVJ**

>NC_055273.1 Rotavirus J strain BO4351/Ms/2014

*GCCACC****ATG***AGTGAAGAGCTGGCATTCACGTTTAAATCAAAAAAATCAAAGAAACTCTCTCCAAAATTAATCCTTCCAGAAAACGACGCGGCATCTACTGTTATTGTTGAAGAGGACGTTAAATCATTGGCCTCAGAATCTGAGAGTTCAGAACATAGCTATGAAAGATACGCAAAAGCTTATGACGATTTTTCAAGAGAGCTCGCAGCATCAGAGTCAAAATCTGTCGATTTTGCAGAAGATTTCGAGGATGTTGAGACTGTTGAGGAGAAACGCAAGGCGCCAAAGGTATTATCAAAGGTACAAAAGCCGATTGTACAAAGTGGAAACGATGGGCTCGAATATAAACTCGCACAGCTCCAACTTCGATTGGAAAAAGTAGAATCGGAAAATAAGGCCAAAACACTGGAATCAGCAATGAATATGGTAATTGCTAACGTCGATAACTTAACTACACCACAGAAGAAAACGCTCCTCCATGCAATAATTTCAACCATGAAG**TGA**

**NSP2-RVJ**

>NC_055266.1 Rotavirus J strain BO4351/Ms/2014

*GCCACC****ATG***GCGCTGAAGGCGAGTCTGGCTGATTTCGTTGTGAAAACAGATGATGGTTACATTCCTTCTGATACAAGCTGTGAAGCACTTGACAGGTTCAACACAAAAGAAGAGAAGGCACTAAAAGATGCGTATTATAAGCAAGGGGCTGATAGAGCTGAAATTAGGAAAAGAATGTTTGTTACTCCAGGATGCAGTAGGAGAATGACACAGCATGGACTAGTGCCAGTGAAAGAATTAAGATCAGGATCAGCGATCCCTTCTGCTCTGAAAAAACTAATTACTAAGTGGCTCCTTGATATGCTCAATGATGAAGATAATGGTGAAACCGCCGAAAAGTACATAATTGACAAGTTTCCGGATGTTCTAATCTCTGCTGATAAACTGTCTCGACTAGCTCAGAGACTGGAAGATGACTCTGATCTAATTCATGAGTCGGTAGGATTTCAAACGCTTGCTGCAGCGGCGTGCGCAATCCCTTCCACTGTCGCAACAGAGGGTAAATGTGAGATTGTTCGAGCAACTGAAGATGCGATAATCGCAAGATTTGATCCAGTTCCAGAACATTTGAACATGGCTAGACATCGTGGAACATTCTTCAAAGCTTTTCCTGTGAACAAAAATGAACCAATGGTGTATGGAGTTAAGGCGCTGTCAGGATTATCAAATCGCGATTTCATAATGTTCCATGGCCATGGACACTTGAGAACTGTTCCATACAATGAGATACCTGATGCGATTAAATCATTTTCAAGGAAACAGAAAGAGGAAATTACCAAAATAATGTCTGATCCAATTGCGAACAACGCTGGGGATAGATTTTTATCAATGTGCAACATGATCTTACAAAGTGAAAAAATTGAAACCATAATTCAAAAGACGATGAAACCAGAAAAGAAGCAG**TAA**

**Nucleotide sequences of chimeric NSP5 proteins used in this study.**

>NSP5∆T/H/TA

gatcacgcgtgccaccATGAGCGACGTGCCTCGATTTGATCTCAGAAGTAAAAGGAAAGTACAAAAGAAGCAGAAAGTTGATATTTTTGACGAAGACGATAAGGAATCATCAATTCAGGTTGACGGTGAATCAGATTCATTAGCTTCAGCGGATACAAGTTCAAATCATAGTTACGAAGATTATTCAAAAGCTTATAAAGATCTAACCTTAGAGACACCAATTGACCAGCCAATTGGAGATGACGATCAAGTTGACTCTGTTTCGACCATTCTTGAAAGTAAGACTAATGATTCGTGGTATAATAAAACTGTTGAAGCTGAAGAACGAATTAAAAAACCGCAAGGGGTAGCTAAACAAAAGATCCAACAAATTGATGAAGATACATCTGATTTAAAGCTTCAACTAGCACAGCTCTCACTAAAAATACAAAAAATGGAATCAGAAACAAAAACCAGAACAGTCGATTCAAAATACTTCGCATTAAGAATGAGAATGAAACAAGTCGCAATGCAATTGATTGAAGATTTGtccggagatc

>NSP5/H/TA

gatcacgcgtgccaccATGAGCGACGTGCCTCGATTTGATCTCAGAAGTAAAAGGAAAGTACAAAAGAAGCAGAAAGTTGATATTTTTGACGAAGACGATAAGGAATCATCAATTCAGGTTGACGGTGAATCAGATTCATTAGCTTCAGCGGATACAAGTTCAAATCATAGTTACGAAGATTATTCAAAAGCTTATAAAGATCTAACCTTAGAGACACCAATTGACCAGCCAATTGGAGATGACGATCAAGTTGACTCTGTTTCGACCATTCTTGAAAGTAAGACTAATGATTCGTGGTATAATAAAACTGTTGAAGCTGAAGAACGAATTAAAAAACCGCAAGGGGTAGCTAAACAAAAGATCCAACAAATTGATGAAGATACATCTGATTTAAAGCTTCAACTAGCACAGCTCTCACTAAAAATACAAAAAATGGAATCAGAAACAAAAACCAGAACAGTCGATTCAGCATATCACACAATTATAACTCAGGCGGAAAATTTAACCACTCCACAGAAAAAATCGTTGATAGCAGCAATAATTGCAACAATGAGAAAATACTTCGCATTAAGAATGAGAATGAAACAAGTCGCAATGCAATTGATTGAAGATTTGtccggagatc

>NSP5∆T/J/TA

gatcacgcgt*gccacc****ATG***AGTGAAGAGCTGGCATTCACGTTTAAATCAAAAAAATCAAAGAAACTCTCTCCAAAATTAATCCTTCCAGAAAACGACGCGGCATCTACTGTTATTGTTGAAGAGGACGTTAAATCATTGGCCTCAGAATCTGAGAGTTCAGAACATAGCTATGAAAGATACGCAAAAGCTTATGACGATTTTTCAAGAGAGCTCGCAGCATCAGAGTCAAAATCTGTCGATTTTGCAGAAGATTTCGAGGATGTTGAGACTGTTGAGGAGAAACGCAAGGCGCCAAAGGTATTATCAAAGGTACAAAAGCCGATTGTACAAAGTGGAAACGATGGGCTCGAATATAAACTCGCACAGCTCCAACTTCGATTGGAAAAAGTAGAATCGGAAAATAAGGCCAAAACACTGGAATCAAAATACTTCGCATTAAGAATGAGAATGAAACAAGTCGCAATGCAATTGATTGAAGATTTGtccggagatc

1. Cloning restriction sites are underlined.
2. Kozak sequence is labeled in italics.
3. The start codon is labeled in bold.

**SUPPLEMENTARY TABLES**

**Table S1.** Pairwise similarity between NSP5 and NSP2 of RVA with RV species B to J.

|  | **Similarity (%) to NSP5/RVA** |  | **Similarity (%) to NSP2/RVA** |
| --- | --- | --- | --- |
| **NSP5/RVA** | 100.00 | **NSP2/RVA** | 100.00 |
| **NSP5/RVB** | 42.99 | **NSP2/RVB** | 34.88 |
| **NSP5/RVC** | 44.29 | **NSP2/RVC** | 56.29 |
| **NSP5/RVD** | 37.79 | **NSP2/RVD** | 60.87 |
| **NSP5/RVF** | 45.54 | **NSP2/RVF** | 53.80 |
| **NSP5/RVG** | 32.72 | **NSP2/RVG** | 34.20 |
| **NSP5/RVH** | 35.85 | **NSP2/RVH** | 35.94 |
| **NSP5/RVI** | 33.66 | **NSP2/RVI** | 33.24 |
| **NSP5/RVJ** | 30.99 | **NSP2/RVJ** | 42.35 |

*Similarity data were obtained by global alignment (Needleman-Wunsch) using matrix BLOSUM62, Gap open penalty of 10.0 and Gap extend penalty of 1.0

**Table S2.** The predicted molecular weight of NSP5 and NSP2 proteins from diverse RV species fused to BAP or HA.

| **RV specie** | **Host** | **Strain** | **NSP5^a^ (kDa)** | **NSP5-BAP^a^**  **(kDa)** | **NSP2^a^ (kDa)** | **NSP2-HA^a^**  **(kDa)** |
| --- | --- | --- | --- | --- | --- | --- |
| **A** | simian | SA11 | 21.72 | 23.7 | 36.56 | 37.7 |
| **B** | human | CAL-1 | 19.77 | 21.7 | 34.52 | 35.6 |
| **C** | porcine | 12R021 | 23.21 | 25.2 | 35.76 | 36.9 |
| **D** | chicken | 05V0049 | 22.26 | 24.2 | 35.88 | 37.0 |
| **F** | chicken | 03V0568 | 24.38 | 26.3 | 36.57 | 37.7 |
| **G** | chicken | 03V0567 | 20.84 | 22.8 | 34.50 | 35.6 |
| **H** | porcine | SP-VC36 | 20.36 | 22.3 | 33.29 | 34.4 |
| **I** | raccoon dog | SD-MO2 | 17.72 | 19.7 | 33.87 | 35.0 |
| **J** | bat | BO4351 | 18.48 | 20.4 | 33.55 | 34.7 |

1. Predicted molecular weight (kDa).

**Table S3.** Oligonucleotides used in this study

| **Amplified DNA segment** | **Oligonucleotide sequence** |
| --- | --- |
| NSP5-BAP/A | Fwd:5’-gatcacgcgtgccacc**atg**tctctcagtattgacgtg-3’  Rev:5’-gatcgcggccgc**tca**ttcgtgccactcaatcttctgtgcttca aagatatcattcagtcctccggacaaatcttcaatcaattgcat-3’ |
| NSP5-BAP/B | Fwd:5’-gatcacgcgtgccacc**atg**gcagaa-3’  Rev:5’-gatcgcggccgc**tca**ttcgtgccactcaatcttctgtgcttca aaga tatcattcagtcctccggagttcatagaattaataattgcgt-3’ |
| NSP5-BAP/C | Fwd:5’-gatcacgcgtgccacc**atg**tctgat-3’  Rev:5’-catgcggccgc**tca**ttcgtgccactcaatcttctgtgcttca aagatatc attcagtcctccggacatttcagcaataatatcaac-3’ |
| NSP5-BAP/G | Fwd:5’-gatcacgcgtgccacc**atg**gctgaa-3’  Rev:5’-gatcgcggccgc**tca**ttcgtgccactcaatcttctgtgcttca aaga tatcattcagtcctccggatttcattgacataactattgc-3’ |
| NSP5-BAP/H | Fwd:5’-gatcacgcgtgccacc**atg**agcgac-3’  Rev:5’-gatcgcggccgc**tca**ttcgtgccactcaatcttctgtgcttca aagatatc attcagtcctccggatctcattgttgcaattattgc-3’ |
| NSP5-BAP/I | Fwd:5’-gatcacgcgtgccacc**atg**gcagac-3’  Rev:5’-gatcgcggccgc**tca**ttcgtgccactcaatcttctgtgcttca aagatatc attcagtcctccggatttcattgaggatgctagcgc-3’ |
| NSP5-BAP/J | Fwd:5’-gatcacgcgtgccacc**atg**agtgaa-3’  Rev:5’-gatcgcggccgc**tca**ttcgtgccactcaatcttctgtgcttca aagatatcattcag tcctccggacttcatggttgaaattattgc-3’ |
| BAP-NSP5/D | Fwd:5’-gatcacgcgtgccacc**atg**ggactgaatgatatctttgaag caca gaagattgagt ggcacgaaatggatgatttagactttaattt-3’  Rev:5’-gatcgcggccgc**tta**ataactttcagc-3’ |
| BAP-NSP5/F | Fwd:5’-gatcacgcgtgccacc**atg**ggactgaatgatatctttgaag cacagaagattgagt ggcacgaaagcatggatcttgatatagact-3’  Rev:5’-gatcgcggccgc**tta**tagatcggatat-3’ |
| NSP5(1-178)-BAP/A | Fwd:5’-gatcacgcgtgccacc**atg**tctctcagtattgacgtg-3’  Rev:5’-agtctccggacttcttatatttacagttctt-3’ |
| NSP5(1-124)-BAP/B | Fwd:5’-gatcacgcgtgccacc**atg**gcagaa-3’  Rev:5’-gatctccggaatttaactctgacaatttttc-3’ |
| NSP5(1-150)-BAP/C | Fwd:5’-gatcacgcgtgccacc**atg**tctgat-3’  Rev:5’-gatctccgga**aat**tcctgccgcaacatgagc-3’ |
| NSP5(1-144)-BAP/G | Fwd:5’-gatcacgcgtgccacc**atg**gctgaa-3’  Rev:5’-gatctccgga**tgc**ttctttaattacatccat-3’ |
| NSP5(1-151)-BAP/H | Fwd:5’-gatcacgcgtgccacc**atg**agcgac-3’  Rev:5’-gatctccgga**tga**atcgactgttctggtttt-3’ |
| NSP5(1-104)-BAP/I | Fwd:5’-gatcacgcgtgccacc**atg**gcagac-3’  Rev:5’-gatctccgga**att**caatgttggatcacaaac-3’ |
| NSP5(1-136)-BAP/J | Fwd:5’-gatcacgcgtgccacc**atg**agtgaa-3’  Rev:5’-gatctccgga**tga**ttccagtgttttggcctt-3’ |
| BAP-NSP5(15-195)/D | Fwd:5’-gatcacgcgtgccacc**atg**ggcctgaacgacatcttcgaggc tcagaaaatcgaatggcacgaagaaatatctttaatttcgtca-3’  Rev:5’-gatcgcggccgc**tta**ataactttcagcaattaaaat-3’ |
| BAP-NSP5(19-219)/F | Fwd:5’-gatcacgcgtgccacc**atg**ggcctgaacgacatcttcgaggc tcagaaaatcgaatggcacgaaataataggaggatctaatact-3’  Rev:5’-gatcgcggccgc**tta**tagatcggatataagcttagc-3’ |
| V5-NSP5/D | Fwd:5’-gatcacgcgtgccacc**atg**ggtaagcctatccctaaccctctc ctcggtctcgattctacgatgatggatgatttagacttt-3’  Rev:5’-gatcgcggccgcttaataactttcagc-3’ |
| V5-NSP5/F | Fwd: 5’-gatcacgcgtgccacc**atg**ggtaagcctatccctaaccctctc ctcggtctcgattctacgatgagcatggatcttgatata-3’  Rev:5’-gatcgcggccgc**tta**tagatcggatat-3’ |
| NSP2-HA/A | Fwd:5’-gatcctcgag**atg**gctgagctagcttgcttt-3’  Rev:5’-gatcgcggccgc**tta**agcgtaatctggaacgtcgtatgggta ggcaacgccaacttgagaaacttc-3’ |
| NSP2-HA/B | Fwd:5’-gatcacgcgtgccacc**atg**acgcaatcagtt-3’  Rev:5’-gatcgcggccgc**tta**agcgtaatctggaacgtcgtatgggta gcttcttttcttttcaaaaat-3’ |
| NSP2-HA/C | Fwd:5’-gatcacgcgtgccacc**atg**gccgag-3’  Rev:5’-gatcgcggccgc**tta**agcgtaatctggaacgtcgtatgggta atttccaaccattgaaattaa-3’ |
| NSP2-HA/D | Fwd:5’-gatcacgcgtgccacc**atg**gcggag-3’  Rev:5’-gatcgcggccgc**tta**agcgtaatctggaacgtcgtatgggta attcacagtggacatttc-3’ |
| NSP2-HA/F | Fwd:5’-gatcacgcgtgccacc**atg**gccgag-3’  Rev:5’-gatcgcggccgc**tta**agcgtaatctggaacgtcgtatgggta atttccgtttataacagacat-3’ |
| NSP2-HA/G | Fwd:5’-gatcacgcgtgccacc**atg**acgcag-3’  Rev:5’-gatcgcggccgc**tta**agcgtaatctggaacgtcgtatgggta ctgtttcttgtcgaatttcat-3’ |
| NSP2-HA/H | Fwd:5’-gatcacgcgtgccacc**atg**tcgatt-3’  Rev:5’-gatcgcggccgc**tta**agcgtaatctggaacgtcgtatgggtat  ttcttctcatgtttcataac-3’ |
| NSP2-HA/I | Fwd:5’-gatcacgcgtgccacc**atg**gcgctg-3’  Rev:5’-gatcgcggccgc**tta**agcgtaatctggaacgtcgtatgggtat  ttctgtgttcttacagtcac-3’ |
| NSP2-HA/J | Fwd:5’-gatcacgcgtgccacc**atg**gcgctg-3’  Rev:5’-gatcgcggccgc**tta**agcgtaatctggaacgtcgtatgggtact  gcttcttttctggtttcat-3’ |

*Restriction sites are underlined.

**Initiation and stop codons are labeled in bold.
